# Molecular Aspects of Methylcadmium Toxicity: Effects on the H_2_O_2_ Reduction by Cysteine and Selenocysteine Disclosed In Silico

**DOI:** 10.1021/acs.chemrestox.5c00409

**Published:** 2025-12-11

**Authors:** Alessandro Rubbi, Francesco Lambertini, Pablo A. Nogara, Marco Bortoli, João B. T. Rocha, Laura Orian

**Affiliations:** † Dipartimento di Scienze Chimiche, 9308 Università Degli Studi di Padova, via Marzolo 1, Padova 35131, Italy; ‡ Federal Institute of Education, Science and Technology Sul-rio-grandense (IFSul), Av. Leonel de Moura Brizola, 2501, Bagé, Rio Grande do Sul 96418-400, Brazil; § Department of Biochemistry and Molecular Biology, Center for Natural and Exact Sciences, 28118 Federal University of Santa Maria, Camobi, Santa Maria 97105-900, Brazil; ∥ Department of Chemistry and Hylleraas Centre for Quantum Molecular Sciences, University of Oslo, P.O. Box 1033, Oslo 0315, Norway

## Abstract

Cadmium (Cd), like the other group 12 elements (Zn and Hg), has a high affinity for sulfur (S) and selenium (Se), a property that strongly influences its adverse biological effects. Although the symptoms of Cd toxicity are diverse, a common denominator is found in oxidative stress, resulting in the disruption of redox balance in cells and the proliferation of reactive oxygen species (ROS) and harmful radicals. Methylcadmium (CH_3_Cd^+^) is a convenient model to study Cd pro-oxidant activity in silico. In this work, the effect of CH_3_Cd^+^ on the peroxy-reducing potential of cysteine (Cys) and selenocysteine (Sec) is investigated at the ZORA-BLYP-D3­(BJ)/TZ2P level and compared to our current knowledge on the analogous molecular aspects of methylmercury’s toxicity (CH_3_Hg^+^). Molecular docking simulations indicate that CH_3_Cd^+^ binds favorably to the catalytic sites of the GPx1 and TrxR1 enzymes. The short distances between the metal and Sec suggest that a nucleophilic attack by Se to Cd leading to the inhibition of the enzyme is indeed possible. Methylcadmium pro-oxidant activity is—if not equal—only slightly inferior to that of methylmercury.

## Graphical abstract

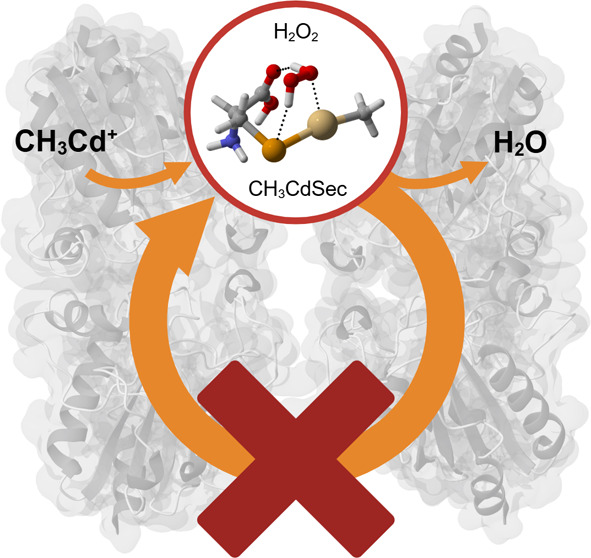

## 1 Introduction

Cadmium (Cd) is a transition metal mostly known for its use alongside nickel in batteries, which is now discontinued. Its discovery is jointly attributed to the Germans Roloff and Stromeyer, who in the early 19th century first found evidence of an unknown oxide impurity in zinc oxide and carbonate samples.^1^ A sibling to zinc (Zn) and mercury (Hg), the element has a great affinity for chalcogens and is predominantly found in small amounts in zinc sulfide minerals.^2^ Being a relatively rare component of the Earth’s crust, it has no biological role: the only known example of a native enzyme incorporating Cd is a carbonic anhydrase found in a marine diatom.^3^ Of note, Cd^2+^ can substitute for Zn^2+^ in certain metalloproteins without significantly altering the catalytic activity of some Zn^2+^-dependent enzymes (e.g., porphobilinogen synthase) or affecting the structural role of Zn^2+^ in proteins such as zinc finger tristetraprolin.^4–7^ But in most cases—particularly in both invertebrates and vertebrates—Cd^2+^ binding to Zn^2+^ binding sites typically leads to a loss in protein function.^8,9^

Cadmium’s toxicity is a cause of great concern in the environmental and medical field. Human exposure to the metal occurs predominantly by disposal of Cd-containing compounds and products.^10^ Nonoccupational causes of intake are inhalation due to air pollution or smoking and ingestion of contaminated foods and drinks.^11,12^ The most severe case of Cd poisoning occurred in Japan during the 1950s, when some inhabitants of the Jinzu river basin were affected by kidney failure and bone softening following pollution of local rice crops from a nearby zinc mine.^13^ A chronic exposure to low amounts of Cd leads to a wide range of equally serious health issues, including kidney and liver damage, osteoporosis, syndromes of the respiratory, cardiovascular, reproductive, and nervous systems, and cancer.^10,11,14,15^

Once in the organism, Cd binds to nucleophilic sites of numerous biomolecules, such as structural, carrier, and channel proteins, enzymes, and transcription factors.^11^ Cd complexes resembling their endogenous counterparts are formed, interfering particularly with Ca, Fe, Zn, and Cu homeostasis. Thiol-/thiolate-bearing biomolecules, such as metallothioneins (MTs), are the most frequent target of Cd binding in vivo. MTs are low-molecular-mass intracellular proteins characterized by a large ratio of cysteine (Cys) residues, ubiquitous among eukaryotic organisms, that bind a variety of metal ions (mainly Zn^2+^ and Cu^+^) through the formation of metal-thiolate clusters. Most importantly, they are the main site of Cd^2+^ accumulation and are responsible for its outstanding biological half-life, which can easily span one or two decades.^16,17^

In general, the biochemistry of Cd is not as well-understood as that of Hg, despite the elements sharing many similarities. One of the main adverse effects of cadmium intake is inflammation, typically linked to oxidative stress (OS).^12,18^ OS arises from an imbalance in cellular redox homeostasis, caused by increased production of reactive oxygen species (ROS) and harmful radicals and reduced antioxidant defense.^18^ In fact, epidemiological studies have linked Cd exposure in humans to increased inflammation and associated pathological processes.^11,19^ Since Cd^2+^ is not redox active, oxidative stress is triggered in indirect ways, such as by interference with redox-regulating processes or by inhibition of antioxidant species.^20^ In mitochondria, which are the major sources of endogenous ROS,^21^ Cd^2+^ uptake leads to a dysfunction of the electron transport chain and membrane permeability.^22^ Treatment with Cd was demonstrated to induce ROS production at complex I flavin and at complex III sites.^23,24^ Additionally, the activity of complexes II, III, and IV was shown in multiple occasions to be significantly diminished by the metal.^24–27^ Glutathione (GSH), an abundant reducing agent in cells, has a high affinity for Cd^2+^, and intracellular accumulation of the metal is known to disrupt the GSH/GSSG redox balance.^28,29^

Catalytic thiols and selenols targeted by Hg, such as Cys or selenocysteine (Sec) residues in glutathione peroxidase (GPx) and in thioredoxin reductase (TrxR), are also likely to coordinate Cd^2+^ with a loss in enzymatic activity.^30,31^ In the catalytic cycle of GPx, the selenol group within its active site is initially oxidized by a hydroperoxide and then reduced by GSH.^32–35^ GPx peroxidatic activity is seemingly negatively affected by Cd intake, but experimental evidence as such is not conclusive.^36–42^ The interaction between Cd^2+^ and the Sec residue in GPx enzymes’ active site, as well as the interaction of the metal with the selenenic form of the residue, remains poorly understood. TrxR can reduce several substrates, such as thioredoxin, hydroperoxides, and disulfide groups,^43^ and is inhibited in neuroblastoma cells and rat erythrocytes cells following Cd exposure, resulting in high OS.^44,45^ Though Cd is expected to have a high affinity for the selenolate of the TrxR Sec residue in the active site, little is known about the nature of their interaction at the molecular level. TrxR activity was also shown not to correlate with blood cadmium levels in human subjects, implying that the metal’s intake does not simply result in a downregulation of the enzyme.^19^ Since enzymes like GPx and TrxR are essential for regulating the redox equilibrium in the cell, any impairment of their function surely contributes to ROS proliferation and has an adverse effect.^46^

(Mono)­methylcadmium (CH_3_Cd^+^) is a possible environmental and biological form of Cd, although there is no clear consensus among researchers. While the relationship between thiol and selenol groups with methylmercury (CH_3_Hg^+^), a major and severely toxic carrier of organic mercury, has been extensively studied,^30^ the literature on CH_3_Cd^+^ is scarcer. The environmental methylation of Cd^2+^ to CH_3_Cd^+^ has been little explored. However, Pongratz and Heumann reported its occurrence at low concentrations—ranging from the detection limit of 470 to more than 700 pg per liter—in seawater and Arctic meltwater.^47^ Despite these picomolar levels, CH_3_Cd^+^ accounted for up to 50% of the total amount of cadmium. The authors went on to demonstrate that CH_3_Cd^+^ can be produced by polar bacteria in the laboratory, possibly by the same that methylate mercury.^48,49^ To the best of our knowledge, the effects of CH_3_CdCl intake have been experimentally probed only once, suggesting it is seriously nephrotoxic.^50^ Similar arguments can be made for dimethylcadmium (Cd­(CH_3_)_2_), for which scientific data are also very limited. Cd­(CH_3_)_2_ is presumed to be even more dangerous than CH_3_Cd^+^, due to its greater volatility and lipophilicity, and to its similarities to dimethylmercury (Hg­(CH_3_)_2_), which is well-known for its extreme toxicity.^51^ The low stability of Cd­(CH_3_)_2_ and Hg­(CH_3_)_2_ in aqueous media and the significant concerns associated with the use of these compounds have contributed to reluctance to study them.^52–54^ Nevertheless, monomethyl species are deemed to be the predominant organic forms of both Cd and Hg from an environmental toxicology perspective.^47,55^ The toxicity of CH_3_Hg^+^, Hg^2+^, and Cd^2+^ is largely dictated by their interactions with abundant low-molecular-mass and high-molecular-mass thiol-containing molecules (e.g., GSH and proteins such as albumin and hemoglobin).^56–59^ Their binding to the much less abundant selenol-containing molecules (selenoproteins) is also expected to be a common denominator of the toxicity of both metals, given the higher affinity of electrophilic Hg and Cd species for –SeH groups compared to that of –SH groups. Still, data on the interaction of CH_3_Cd^+^ with these two biologically relevant functional groups are not available.

Computational methods are a valuable tool to expand the knowledge of molecular aspects of Cd reactivity with Cys and Sec. Recent Density Functional Theory (DFT) calculations (level of theory: ZORA-BLYP-D3­(BJ)/TZ2P) have provided valuable insight into methylmercury chalcogenolate chemistry, performing rather well in comparison to ab initio methods and crystallographic data.^60–62^ In this work, the effect of CH_3_Cd^+^ binding on the peroxidatic potential of Cys and Sec has been studied at the ZORA-BLYP-D3­(BJ)/TZ2P level of theory, modeling the reactions of normal and toxified (i.e., CH_3_Cd^+^-substituted) residues with H_2_O_2_ (Schemes 1 and 2). Here, CH_3_Cd^+^ serves a dual purpose: in addition to exploring the effect of organic Cd on molecular models of the enzymes, it allows for a meaningful comparison with data on methylmercury.^60^ The effects of both implicit and explicit H_2_O solvation have also been assessed. Activation Strain Analysis (ASA) has been performed to gain qualitative and quantitative insight into the chemistry of CH_3_Cd^+^ from a molecular orbital (MO) perspective and to rationalize its toxicity. Finally, molecular docking simulations were carried out to evaluate the intermolecular interactions between the catalytic sites of GPx1 and TrxR1 with CH_3_Cd^+^ and its derivatives.

**Scheme 1:**
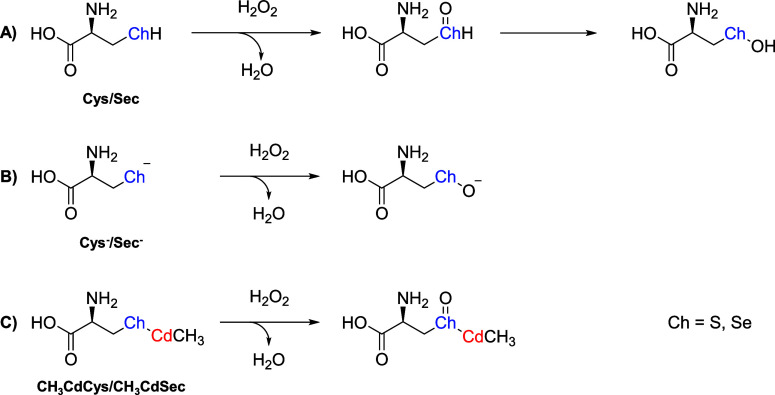
Oxidation of Cys/Sec (A), Cys^–^/Sec^–^ (B), and CH_3_Cys/CH_3_CdSec (C) by H_2_O_2_ (Ch = S for Cys and Se for Sec)

**Scheme 2:**
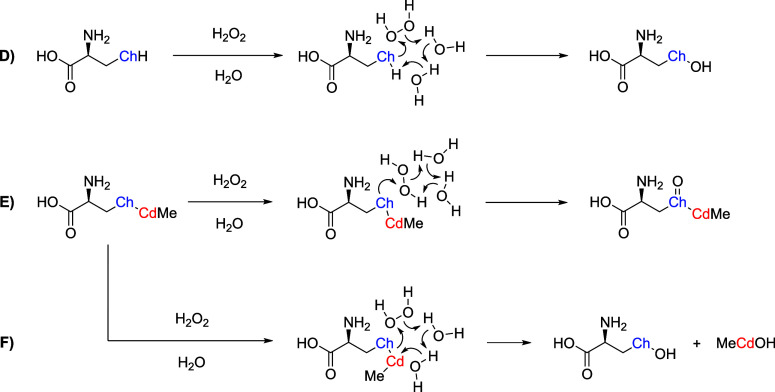
SAPE Mechanism for the Oxidation of Cys and Sec (D) and of CH_3_CdCys and CH_3_CdSec (E,F) by H_2_O_2_, Featuring Two Explicit H_2_O Molecules (Ch = S, Se)^a^

a The pathway leading to the formation of chalcogenic acid and CH_3_CdOH (F) is preferred over the path leading to the chalcogenoxide (i.e., sulfenate-κO and selenenate-κO) product (E)

## 2 Computational Methods

All DFT calculations were performed using the Amsterdam Density Functional (ADF 2019.307) and the Amsterdam Modeling Suite (AMS 2020.103) programs.^63–65^ Geometry optimizations were carried out using the GGA exchange functional BLYP,^66^ with Grimme’s DFT-D3 dispersion and Becke–Johnson damping corrections.^67–72^ Zeroth order regular approximation (ZORA) has been applied for all elements to account for scalar relativistic effects,^73–75^ along with the TZ2P basis set.^76^ This is a large set of relativistic Slater-type functions of triple-ζ quality augmented with two sets of polarization functions per atom, designed for calculations with the ZORA formalism. The small frozen core approximation was also employed, excluding core electrons from the SCF optimization, e.g., up to 1s for C, 2p for S, 3p for Se, and 3d for Cd.^76^ Solvent-assisted proton exchange (SAPE) has been used to model proton transfer reactions (Scheme 2). All structures met tight convergence criteria, with a maximum tolerance on the largest Cartesian nuclear gradient of 1 × 10^–5^ Hartree/Å (keyword “grad” in ADF). Numerical integration and density fitting quality were set to “verygood” (parameters “beckegrid” and “zlmfit”), while the other settings were left to their default values. Analytical frequencies were computed at the same level of theory of the optimization procedure to verify that all minima display only real, positive frequencies and that a single imaginary frequency is associated with each transition state (TS).^77–79^ This level of theory, which is referred to as ZORA-BLYP-D3­(BJ)/TZ2P, has been benchmarked against higher-rung methods and has been extensively applied to investigate metal–organochalcogen chemistry.^60–62^ Intrinsic Reaction Coordinate (IRC) calculations were carried out to determine, for each TS, the potential energy surface (PES) path connecting the corresponding reactant and product complex (RC and PC).^80^

Activation Strain and Energy Decomposition Analysis (ASA/EDA) have been performed on IRC paths in order to gain insight on the differences in the energy barriers.^81–85^ ASA is a fragment-based approach for the systematic study of bonding and reactivity in chemical reactions. Within this framework, the total energy change (Δ*E*) along a chosen reaction coordinate (ζ) on the PES is decomposed as the sum of the strain energy (Δ*E*_strain_) and the interaction energy (Δ*E*_int_) (eq 1).

1 $$\Delta E\left(\zeta \right)=\Delta E_{strain}\left(\zeta \right)+\Delta E_{\mathrm{int}}\left(\zeta \right)$$

The strain energy accounts for the structural distortion of the noninteracting reactant fragments from their respective equilibrium geometries, as they progress along the reaction pathway and approach the TS conformation. It arises from the rigidity of the reactants, and it reflects, for each fragment, the extent of bond stretching (or breaking) and angular deformations. Conversely, Δ*E*_int_ represents the interaction between the distorted fragments and depends on their electronic structure and mutual spatial arrangement. The interplay between these two quantities defines the reaction barrier height. The interaction term can be further analyzed through EDA, providing quantitative insights into bonding (eq 2).^86^ Δ*E*_int_ is decomposed into three main contributions: classical electrostatic interaction (Δ*V*_elstat_), Pauli repulsion (Δ*E*_Pauli_), and orbital interaction (Δ*E*_oi_). Δ*V*_elstat_ is defined as the interaction energy between the unperturbed charge distribution of the fragments, i.e., the sum of nuclei repulsion, nucleus–electron density attraction, and electron-densities repulsion. Δ*E*_Pauli_ is the energy increase arising from same-spin electrons avoiding each other and increasing in kinetic energy, which is generally responsible for steric effects.^86^ Finally, orbital interactions account for charge transfer (the mixing of occupied and virtual orbitals, such as HOMO–LUMO interactions) and polarization effects. If dispersion corrections are explicitly included at the level of theory of the EDA calculation, as in this work, their contribution (Δ*E*_disp_) is also included in the interaction energy.

2 $$\Delta E_{\mathrm{int}}\left(\zeta \right)=\Delta V_{elstat}\left(\zeta \right)+\Delta E_{Pauli}\left(\zeta \right)+\Delta E_{oi}\left(\zeta \right)+\Delta E_{disp}\left(\zeta \right)$$

When their effect is critical, orbital interaction contributions are rationalized using Natural Orbitals for Chemical Valence (NOCVs), according to the extended transition state (ETS) NOCV method by Mitoraj et al.^87^

Implicit solvation effects in H_2_O have been evaluated by means of single-point calculations on gas-phase geometries, based on the COnductor-like Screening MOdel (COSMO).^88^ Statistical thermodynamic corrections have been derived from vibrational frequency calculations at the same level of theory of the optimization procedure to compute standard state Gibbs free energies (298.15 K, 1 atm). TS energies (relative to the free reactants) follow the same trends in electronic, Gibbs, and COSMO energies (Tables S1–S4 in the Supporting Information). Therefore, only electronic energies at the ZORA-BLYP-D3­(BJ)/TZ2P level of theory are discussed in this manuscript, to keep consistency with ASA and EDA. Molecular structures and NOCVs were illustrated using visualization tools of CYLview20 and ADF software, respectively.^64,89^

The AutoDock Vina program was used in the docking simulations.^90^ The 3D structures of GPx1 and TrxR1, both as dimers, were obtained from the Protein Data Bank (PDB) with the codes 1GP1 and 2J3N, respectively. Two molecular docking protocols were applied: rigid protein and flexible ligand (rigid–flexible) and semiflexible protein and flexible ligand (semiflexible–flexible). The flexible side chains of some residues (GPx1: Sec45, His79, and Asn82; TrxR1: Cys497 and Sec498) were selected based on their proximity to the ligands observed in the rigid–flexible docking simulation. The grid box of 15 Å^3^ was centered on the selenium atom of the enzymes. Initially, the structures of the enzymes were submitted to molecular dynamics simulations (MD) to obtain a more realistic protein conformation. First, the initial enzyme structures were solvated with TIP3P water (21495 water molecules for GPx1 and 33566 for TrxR1) in a truncated octahedral box. Afterward, the systems were minimized, heated to the target temperature (298.15 K) with a constant volume constraint for 400 ps, and equilibrated for 4 ns at a constant temperature and pressure (1 bar). The MD simulations were carried out for 500 ns in the same conditions of the equilibration using the Langevin thermostat and a Monte Carlo barostat. The nonstandard AMBER ff14sb parameters for Sec were taken from the literature.^91^ The structures of the ligands (CH_3_Cd^+^, CH_3_CdOH, and CH_3_CdCys) were obtained by DFT calculations, as described above. Hirshfeld partial charges were used for the ligands in the docking simulations.^92^ As in Vina there are no parameters for Cd, the metal center was replaced with Zn.^93,94^ However, the charges and geometries were those obtained from quantum mechanics optimization. Due to the correlation between the number of heavy atoms (N) in the ligands and the predicted binding energies,^95^ the latter were normalized using the size-independent ligand efficiency (SILE) score, SILE = −(D/N^0.3^), where D is the docking binding energy score.^96^ The ligand conformers with the lowest (most negative) binding energy and with the best Se···Cd orientation were selected as the model of the interaction.

## 3 Results and Discussion

### 3.1 Stepwise Oxidation Mechanisms

The mechanism by which the amino acids react directly with H_2_O_2_ is described in Scheme 1. In the case of neutral amino acids Cys and Sec (Scheme 1A), the chalcogenols are initially oxidized to a chalcogenoxide intermediate, which subsequently undergoes isomerization to yield the corresponding chalcogenic acids. The conjugate bases Cys^–^ and Sec^–^ can be oxidized via an anionic mechanism which has been reported for other chalcogenolates, forming the corresponding sulfenate/seleninate products (Scheme 1B).^97–99^ This pathway has been addressed too, since at physiological pH and in the active site of peroxidatic enzymes, selenocysteine and cysteine are in equilibrium with their deprotonated forms. The replacement of H- with a CH_3_Cd-fragment (CH_3_CdCys/CH_3_) allows for the evaluation of the effect of CH_3_Cd^+^ binding on the energetics, by comparison with the two previous cases (Scheme 1C).

Stationary points are illustrated in Figure 1. In the gas phase, the reactants are initially stabilized through the formation of a reactant complex (RC); similarly, after the transition state (TS), a product complex (PC) is found on the PES at an energy lower than that of the free products. The transition states for the isomerization of the oxidized amino acids to chalcogenic acids (**Cys-TS**_**iso**_ and **Sec-TS**_**iso**_) are included for the sake of completeness, as they are only relevant to the gas-phase PES. All reactions are largely exergonic (Table 1), although the products of CH_3_CdCys and CH_3_CdSec oxidation are substantially less stable. Negatively charged chalcogenolates exhibit the lowest activation energies. CH_3_Cd-substituted amino acids display a lower TS energy compared to their nontoxified neutral analogues, although energy barriers are seemingly higher for CH_3_Cd-compounds due to more stabilized RCs (**CH_3_CdCys-RC** and **CH_3_CdSec-RC**). As such, CH_3_CdCys and CH_3_CdSec react more easily with H_2_O_2_ in comparison to Cys and Sec, respectively, yet significantly less easily compared to Cys^–^ and Sec^**–**^. All Se species are more prone to oxidation compared with their S analogues. Methylcadmium affects the energetics in a similar way to methylmercury: the TS energies of CH_3_Cd^+^-substituted amino acids (14.1 and 10.9 kcal·mol^–1^ for CH_3_CdCys and CH_**3**_CdSec, respectively) are slightly more positive than those reported for CH_3_Hg^+^-substituted analogues in a previous work by Madabeni and co-workers, at the same level of theory (12.8 and 9.8 kcal·mol^–1^ for CH_3_HgCys and CH_3_HgSec, respectively).^60^

**Figure 1:**
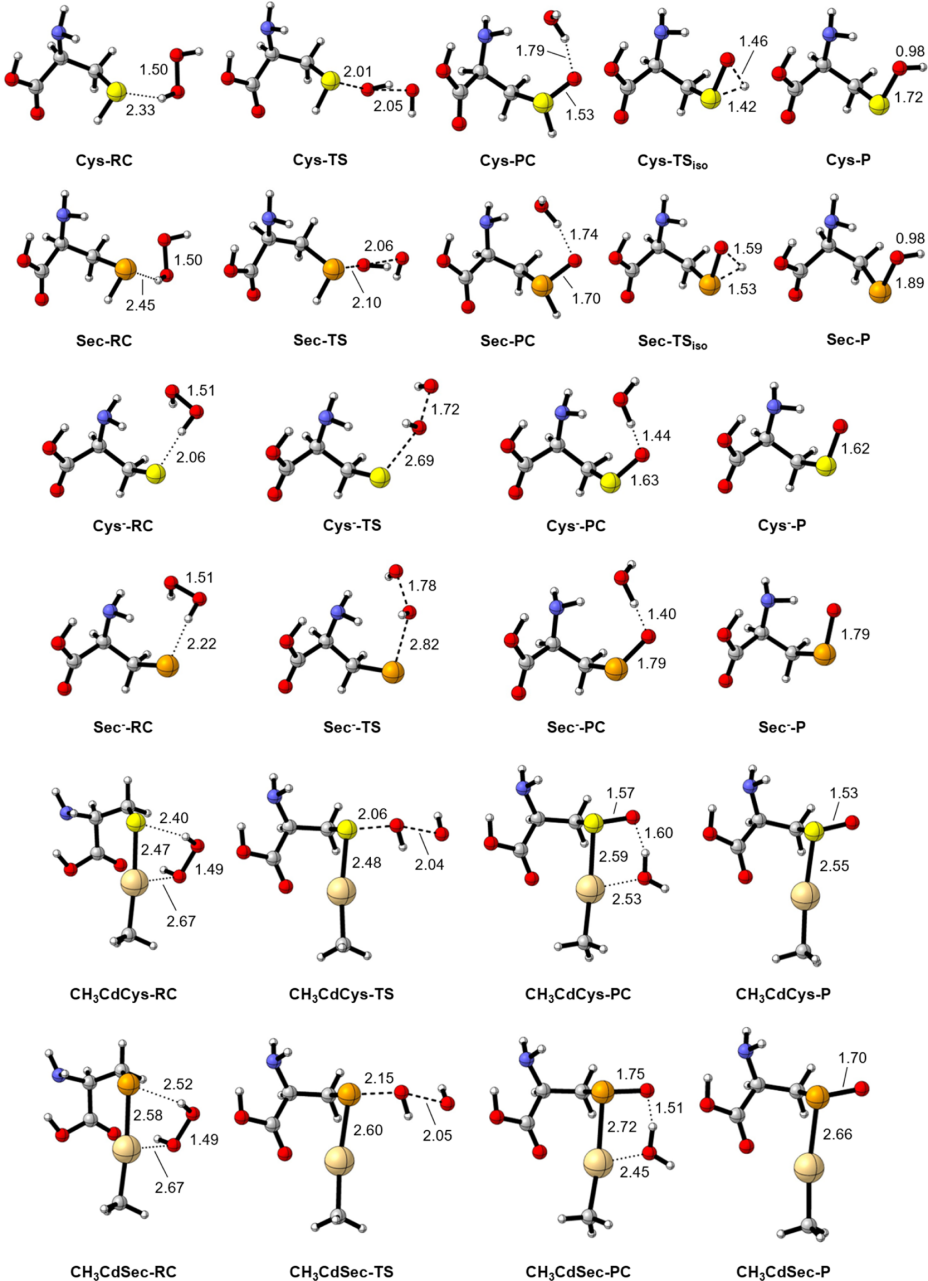
Stationary points for the oxidation of Cys, Sec, CH_3_CdCys, CH_3_CdSec, Cys^–^, and Sec^–^ by H_2_O_2_ (yellow = S, orange = Se, light gold = Cd). All interatomic distances are in Å. Level of theory: ZORA-BLYP-D3­(BJ)/TZ2P.

**Table 1:** Electronic Energies (kcal·mol^–1^) Relative to Free Reactants of Stationary Points for the Oxidation of Cys, Sec, Cys^–^, Sec^–^, CH_3_CdCys, and CH_3_CdSec by H_2_O_2_ ^a^ ^b^

	RC	TS	PC	TS_iso_	P
**Cys**	–6.0	17.8 (23.9)	–47.9	1.0 (48.8)	–49.8
**Sec**	–6.0	14.1 (20.1)	–40.0	1.1 (41.2)	–55.4
**Cys** ^**–**^	–19.9	–13.0 (6.9)	–65.1		–50.2
**Sec** ^**–**^	–18.7	–13.7 (5.0)	–50.3		–44.1
**CH**_**3**_ **CdCys**	–11.6	14.1 (25.8)	–49.2		–31.8
**CH**_**3**_ **CdSec**	–11.9	10.9 (22.9)	–42.5		–22.6

a Activation energies for the oxidation (relative to the RC) and for the isomerization (relative to the PC) are given in parentheses.

b Level of theory: ZORA-BLYP-D3­(BJ)/TZ2P.

### 3.2 SAPE Oxidation Mechanism

The SAPE strategy has been extensively applied to mechanistic studies in organochalcogen chemistry,^100–102^ including the oxidation of methylmercury chalcogenolates,^60^ to accurately model proton transfer reactions. As for the reactions studied in this work, the inclusion of explicit H_2_O molecules is a justifiable choice since a few are typically present within enzymatic pockets. According to the SAPE scheme, H_2_O_2_ is reduced through a concerted proton shuttling mechanism through a network of hydrogen-bonded molecules (Scheme 2). Two H_2_O molecules were found to be sufficient to correctly estimate energy barriers in similar chalcogenide/chalcogenolate systems.^100,103,104^

A comparison of the SAPE stationary points between neutral and toxified residues reveals that the presence of the metal leads to different products, deviating from the stepwise oxidation mechanism (Figure 2). The reactions of Cys and Sec are consistent with those of the minimal systems. At both transition states (**Cys-TS**_**SAPE**_/**Sec-TS**_**SAPE**_), the proton on the chalcogen is being transferred to the closest H_2_O molecule, forming the corresponding chalcogenic acid; notably, no isomerization step is required (Scheme 2D). In the case of CH_3_CdCys and CH_3_CdSec, the metal is involved in the hydrogen-bonded network at both the RC and the TS. The solvent molecules simultaneously assist the H_2_O_2_ reduction and the deprotonation of the H_2_O molecule that is closest to the metal (Scheme 2F). This concerted mechanism results in the formation of chalcogenic acid and methylcadmium hydroxide (CH_3_CdOH), which is preferred over the formation of the chalcogenoxide product (Scheme 2E). Attempts to avoid this nucleophilic substitution at the metal center were unsuccessful. Both Cd–S and Cd–Se bonds are preferentially broken in favor of the formation of the Cd–O bond. This shows some analogies with the reduction of H_2_O_2_ by HgCl^+^ and CdCl^+^ phenylselenolates, for which Zeppilli et al. reported a similar mechanism change when Hg is replaced by Cd.^105^ According to their calculations, the gas-phase reaction of PhSeHgCl with H_2_O_2_ yields the corresponding selenenate-κO (a chalcogenoxide) and H_2_O. In contrast, in the case of PhSeCdCl, the addition product is formed in which two hydroxyl groups are bonded to Cd and Se, respectively. Although they did not make use of explicit solvation, their results ruled out the possibility that ClHgOH is released by hydrolysis of PhSeOHgCl, whereas the formation of ClCdOH in solution was deemed plausible.

**Figure 2:**
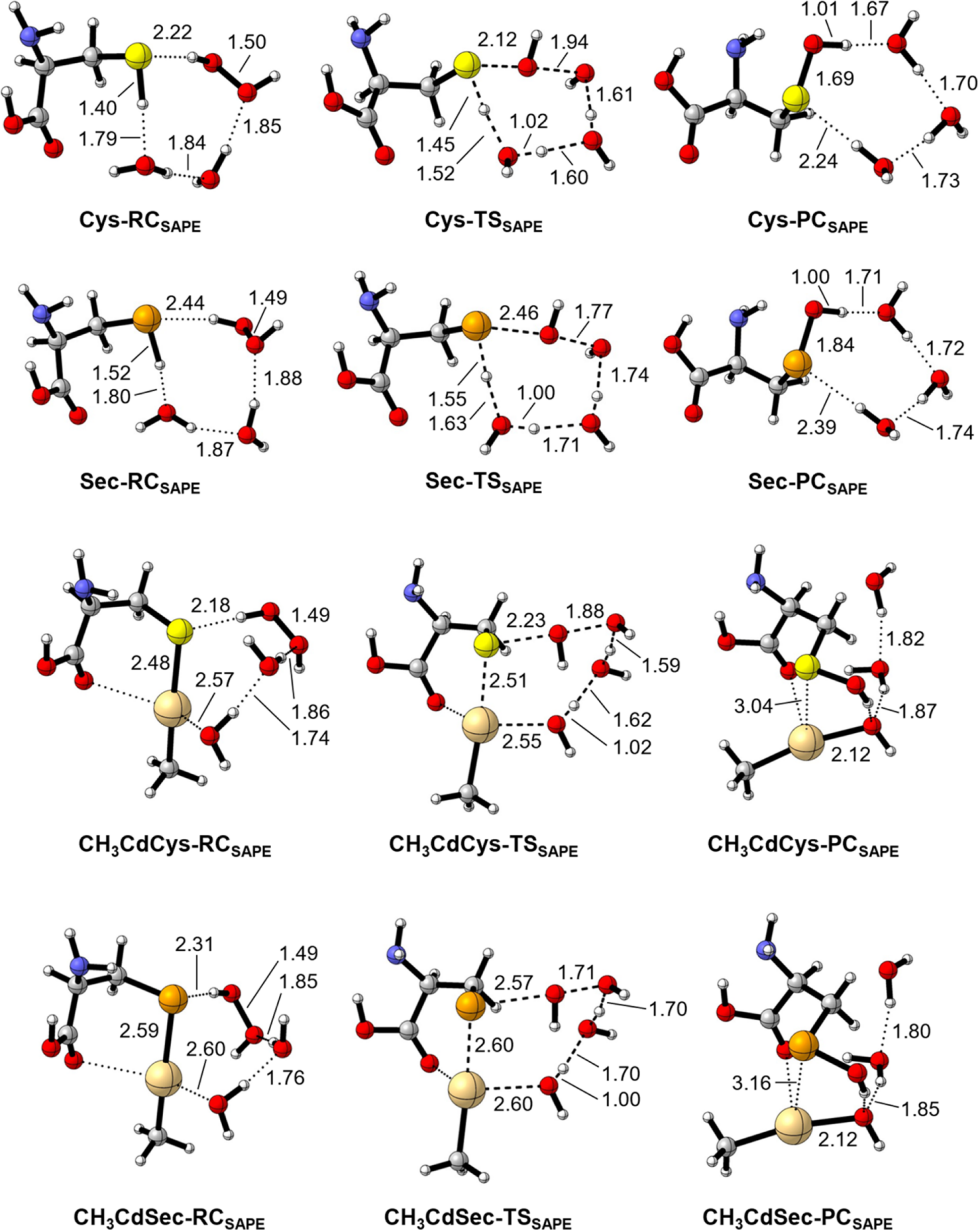
SAPE stationary points for the oxidation of Cys, Sec, CH_3_CdCys, and CH_3_CdSec by H_2_O_2_, featuring two explicit H_2_O molecules (yellow = S, orange = Se, light gold = Cd). All interatomic distances are in Å. Level of theory: ZORA-BLYP-D3­(BJ)/TZ2P.

All reactions are largely exergonic (Table 2). The inclusion of explicit H_2_O molecules lowers the energy barriers by ∼12 kcal mol^–1^ in the case of Cys and Sec. Despite the mechanism change, the activation energies for CH_3_CdCys and CH_3_CdSec are the lowest overall. CH_3_Cd^+^-substituted amino acids are more prone to be oxidized by H_2_O_2_ compared to Cys and Sec, in agreement with the analogous reactions involving methylmercury chalcogenolates. SAPE energy barriers for CH_3_Cd^+^-substituted residues (7.4 and 4.3 kcal·mol^–1^ for CH_3_CdCys and CH_3_CdSec, respectively) are only slightly higher compared to those for CH_3_Hg^+^-substituted amino acids (7.3 and 3.7 kcal·mol^–1^ for CH_3_HgCys and CH_3_HgSec, respectively, reported by Madabeni et al. at the same level of theory).^60^ Since the binding constants of CH_3_Hg^+^ with –SH groups are lower than those of Hg^2+^ and the constants for Cd^2+^ with either –SH and –SeH groups are lower than those of Hg^2+^, we can predict that the affinity of CH_3_Cd^+^ for –SH and –SeH groups will be lower than that of CH_3_Hg^+^.^106–108^ However, metal–chalcogen bond cleavage is not observed for CH_3_Hg^+^-substituted amino acids. This implies that the toxicity of organocadmium species toward these residues is mechanistically different from that of mercury. CH_3_CdCys and CH_3_CdSec are more susceptible to oxidation by H_2_O_2_ compared to (neutral) Cys and Sec. Most notably, their reaction with hydroperoxides releases the metal back in solution, which may potentially bind to other chalcogenolates. CH_3_Cd^+^, unlike CH_3_Hg^+^, would therefore be able to inactivate and promote the oxidation of multiple thiol groups in a “residue-hopping” mechanism. Chelation of Cd^2+^ ions by Cys-rich MTs likely interrupts this chain of reactions: recent experiments on Cd­(II)-bound MTs indicate that such metal–thiolate clusters are resilient to oxidative stress and remain metalated to a significant degree.^109^

**Table 2:** Electronic Energies (in kcal·mol^–1^) Relative to Free Reactants of SAPE Stationary Points for the Oxidation of Cys, Sec, CH_3_CdCys, and CH_3_CdSec by H_2_O_2_. Activation Energies for the Oxidation Relative to the RC Are Given in Parentheses^a^

	RC	TS	PC
**Cys**	–25.3	–12.7 (12.6)	–80.8
**Sec**	–22.4	–15.7 (6.8)	–84.9
**CH**_**3**_ **CdCys**	–27.7	–20.3 (7.4)	–72.4
**CH**_**3**_ **CdSec**	–26.8	–22.6 (4.3)	–70.3

a Level of theory: ZORA-BLYP-D3­(BJ)/TZ2P

### 3.3 Activation Strain and Energy Decomposition Analysis

ASA and EDA have been performed along the IRC path of the oxidation of Cys, Sec, CH_3_CdCys, and CH_3_CdSec by H_2_O_2_, by partitioning them into the amino acid and the hydroperoxide fragments, respectively. The chalcogen–oxygen distance (*d*_Ch‑O_) has been chosen as the reaction coordinate since it allows a consistent case-by-case comparison of interaction terms, i.e., at precise distances between the fragments. The effect of CH_3_Cd^+^ substitution has been scrutinized by separately comparing CH_3_CdCys to Cys and CH_3_CdSec to Sec (Figure 3). Nonetheless, energy profiles of Sec systems resemble those of Cys systems, meaning that the effect of methylcadmium binding on the reaction with H_2_O_2_ does not change much when sulfur is replaced by selenium. All reactions are interaction-controlled, whereas strain, which arises almost exclusively from the dissociation of the peroxide bond (see Table S5 in the Supporting Information), follows the opposite trend. With that being the case, interaction energies were decomposed according to the EDA scheme (Figure 3). Dispersion contributions were found to be negligible for the purpose of this analysis (see Table S6 in the Supporting Information). When comparing Cd-substituted and nonmetalated residues, both Pauli repulsion and electrostatic interaction terms are almost identical in the two cases along the whole reaction coordinate, suggesting that none of the two significantly affects the energy barriers. Conversely, orbital interactions are more negative for CH_3_CdCys and CH_3_CdSec, steering the reactivity toward the oxidation of the toxified amino acids.

**Figure 3:**
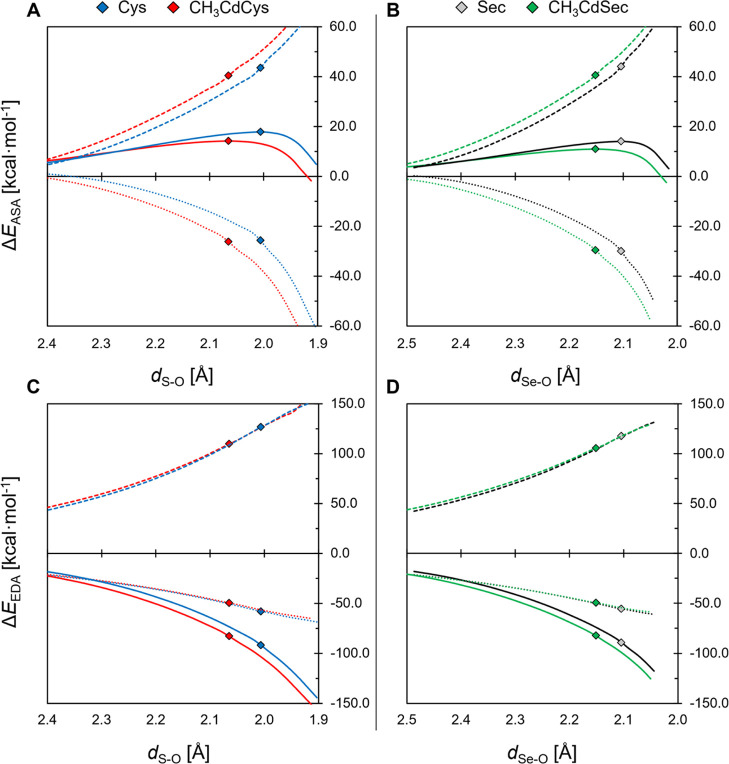
ASA (A,B) and EDA (C,D) plots comparing the oxidation of Cys (blue), CH_3_CdCys (red), Sec (black), and CH_3_CdSec (green) by H_2_O_2_; the reaction coordinate is the chalcogen–oxygen distance (in Å). ASA terms are total energy (solid line), strain (dashed line), and interaction (dotted line); EDA terms are orbital interaction (solid line), Pauli repulsion (dashed line), and electrostatic interaction (dotted line). Dispersion terms are not appreciable at this scale and are not shown. TS points are indicated by markers. Level of theory: ZORA-BLYP-D3­(BJ)/TZ2P.

### 3.4 Orbital Analysis

Having established that orbital interactions play a key role in determining the height of the energy barrier, their contributions were further decomposed according to the ETS-NOCV scheme. The analysis has been performed at consistent points along the *d*_Ch‑O_ coordinate to allow for comparisons among all four reactions (Table 3). In all cases, a single NOCV term accounts for most of the orbital interaction energy (see Figure S1 in the Supporting Information). These stabilizing interactions are associated with the charge transfer from the HOMO of the amino acidic fragment to the LUMO of the H_2_O_2_ fragment, due to orbital overlap during the reaction (Figure 4). Electronic density is donated by a lone-pair p orbital localized on the chalcogen atom, while the σ*_O–O_ antibonding orbital behaves as the acceptor, in line with the reductant and oxidant roles in the reaction, respectively.^110^ Interestingly, there is a clear correlation between the value of Δ*E*_OI_ and the HOMO–LUMO energy gap, meaning that the most negative orbital interaction terms are associated with a more positive chalcogenolate HOMO (relative to the other fragment’s LUMO). The effect of CH_3_Cd^+^ binding is also similar for Cys and Sec: Δ*E*_LUMO–HOMO_ grows by 18–21 kcal·mol^–1^ and, in turn, Δ*E*_OI_ is increased in value by ∼6 kcal·mol^–1^. CH_3_CdCys and CH_3_CdSec higher-energy HOMOs make them better electron donors compared to the neutral amino acids, lowering the energy barrier for the oxidation by H_2_O_2_. This conclusion nicely matches the interpretation proposed in the case of CH_3_Hg^+^-toxified Cys and Sec.^60^ The marginally higher activation energies predicted for CH_3_Cd^+^ over CH_3_Hg^+^ are likely influenced by the less diffuse and polarizable 4d orbitals in Cd in contrast to the Hg 5d orbitals, which may affect the stabilization of the transition state.

**Table 3:** ETS-NOCV Analysis and HOMO–LUMO Energies (in kcal·mol^–1^) of **Cys**, **Sec**, **CH**_**3**_ **CdCys**, and **CH**_**3**_ **CdSec** at *d*_Ch‑O_ ∼2.15 Å. Level of Theory: ZORA-BLYP-D3­(BJ)/TZ2P

	Δ*E*_TS_ ^a^	Δ*E*_OI_ ^b^	Δ*E*_OI,max_ ^c^	Δ*E*_LUMO–HOMO_ ^d^	ν_max_ ^e^
**Cys**	17.9	–52.9	–44.3	–0.1	0.77
**Sec**	14.1	–76.9	–65.9	–23.9	0.92
**CH**_**3**_ **CdCys**	14.2	–58.6	–50.4	–21.2	0.83
**CH**_**3**_ **CdSec**	10.9	–82.4	–72.1	–41.8	0.97

a TS energy from Table 1.

b Orbital interaction from ASA.

c Largest NOCV contribution to Δ*E*_OI_.

d HOMO–LUMO energy difference (Δ*E*_LUMO–HOMO_ = *E* ^A^_LUMO_ – *E* ^D^_HOMO_; A = acceptor, D = donor).

e NOCV eigenvalue of Δ*E*_OI,max_.

**Figure 4:**
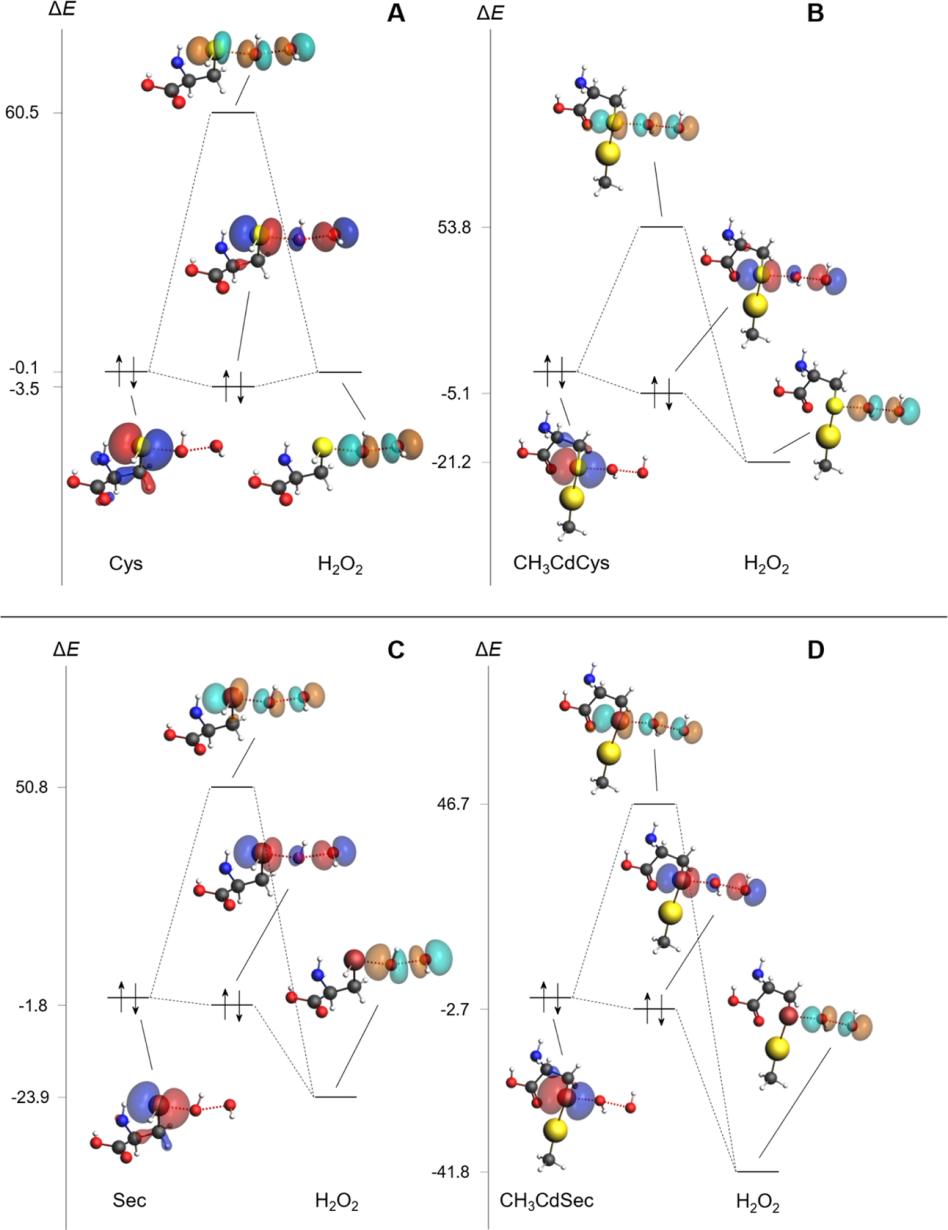
HOMO–LUMO orbital interaction diagram of Cys (A), CH_3_CdCys (B), Sec (C), and CH_3_CdSec (D) at consistent points along the reaction coordinate (*d*_Ch‑O_ ∼2.15 Å). Occupied MOs are colored in blue and red; virtual MOs in cyan and orange (ρ > 0.05). Relative energies (in kcal·mol^–1^) are referred to the chalcogenolate’s HOMO. Level of theory: ZORA-BLYP-D3­(BJ)/TZ2P.

### 3.5 Molecular Docking Simulations

To gain insight into the Cd interaction with selenoenzymes, the CH_3_Cd^+^, CH_3_CdOH, and CH_3_CdCys compounds (putative molecules in the biological medium) were inserted into the catalytic pockets of GPx1 and TrxR1 using molecular docking simulations. Two protocols were used, i.e., one with rigid protein and flexible ligand (rigid–flexible) and the latter with semiflexible protein and another with flexible ligand (semiflexible–flexible). According to the predicted affinities (Figures 5 and 6), CH_3_Cd^+^, CH_3_CdOH, and CH_3_CdCys can all bind to the active site of GPx1 and TrxR1 with favorable thermodynamics (Table 4), interacting with the Sec residue. In both proteins, CH_3_CdCys exhibits the most negative Δ*G* values and the highest SILE score, indicating a more stable complex. However, CH_3_Cd^+^ shows the shortest Se···Cd distance with the selenocysteinyl residue.

**Figure 5:**
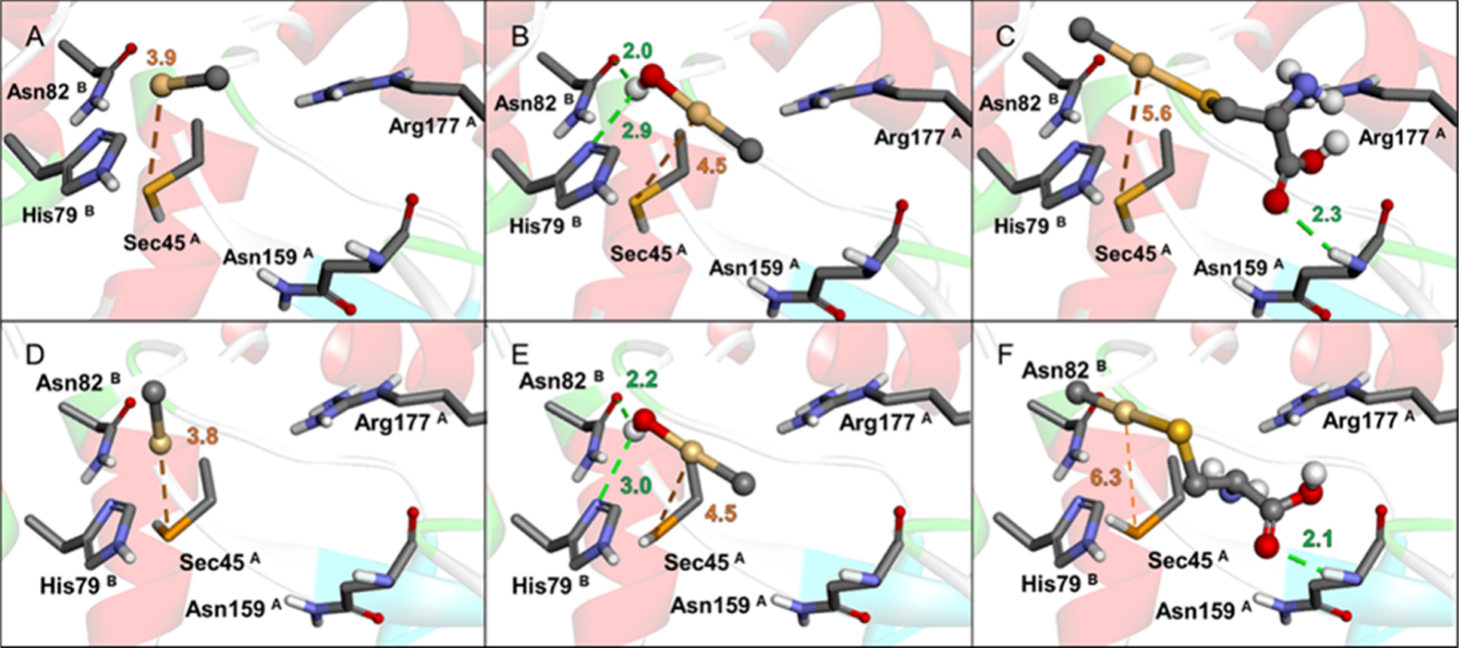
GPx1 docking simulations. Rigid*–*flexible molecular docking with CH_3_Cd^+^ (A), CH_3_CdOH (B), and CH_3_CdCys (C). Semiflexible*–*flexible molecular docking with CH_3_Cd^+^ (D), CH_3_CdOH (E), and CH_3_CdCys (F). All interatomic distances are in Å.

**Figure 6:**
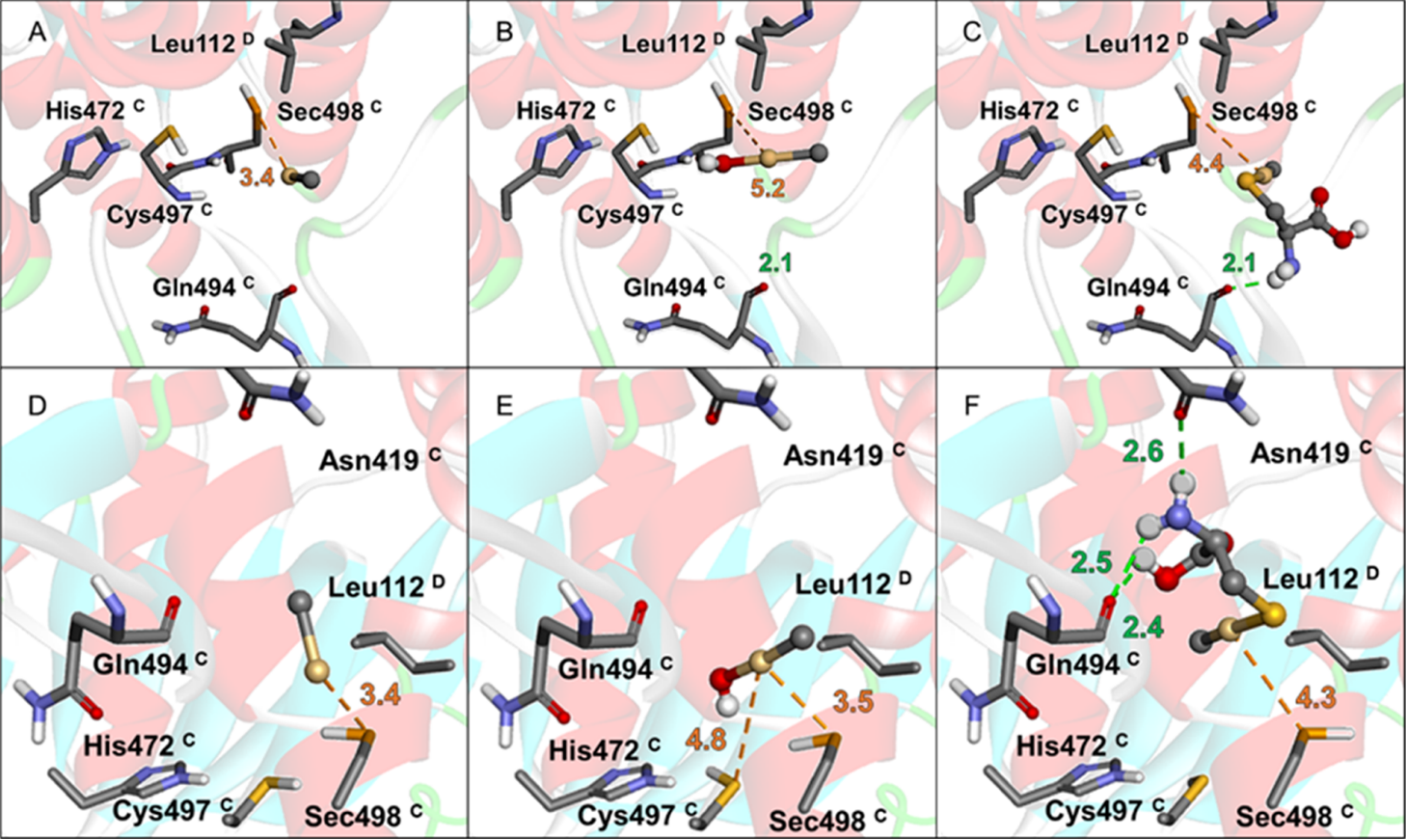
TrxR1 docking simulations. Rigid*–*flexible molecular docking with CH_3_Cd^+^ (A), CH_3_CdOH (B), and CH_3_CdCys (C). Semiflexible*–*flexible molecular docking with CH_3_Cd^+^ (D), CH_3_CdOH (E), and CH_3_CdCys (F). All interatomic distances are in Å.

**Table 4:** Ligand Binding Energies (Δ*G*, kcal·mol^–1^) and Size-Independent Ligand Efficiencies (SILE)^a^

		rigid–flexible			semiflexible–flexible		
**enzyme**	**ligand**	**Δ** *G*	**N**	**SILE**	**Δ** *G*	**N**	**SILE**
**GPx1**	CH_3_Cd^+^	–1.3	2	1.056	–1.4	2	1.137
	CH_3_CdOH	–1.9	3	1.366	–2.5	3	1.798
	CH_3_CdCys	–3.5	9	1.810	–3.5	9	1.810
**TrxR1**	CH_3_Cd^+^	–1.1	2	0.893	–0.3	2	0.244
	CH_3_CdOH	–1.5	3	1.079	–1.0	3	0.719
	CH_3_CdCys	–3.0	9	1.552	–2.3	9	1.190

a N: number of heavy atoms in the ligand; SILE = –(D/N^0.3^); D: docking binding energy score (Δ*G*).

In GPx1, both molecular docking protocols provide analogous results (Figure 5), with CH_3_Cd^+^ and CH_3_CdOH presenting similar binding poses and Se···Cd interaction distances ranging from 3.8 Å to 4.5 Å. In addition, the hydroxyl moiety of CH_3_CdOH can form hydrogen bonds with His79 and Asn82. The CH_3_Cd moiety of CH_3_CdCys shows a Se···Cd distance of 5.6–6.3 Å, and this species can interact with the Asn159 backbone via H-bonding.

In TrxR1, all three ligands interact with Sec498; however, the rigid–flexible and the semiflexible–flexible docking protocols reveal some differences in the conformations of the species (Figure 6). CH_3_Cd^+^ and CH_3_CdOH present similar binding poses, interacting with Sec498 via Se···Cd (3.4–5.2 Å). CH_3_CdCys interacts via H-bonding with the Gln494 backbone, and a Se···Cd interaction at 4.4 Å is observed too in the rigid–flexible simulation. CH_3_CdOH can also interact with Cys497. Using the semiflexible–flexible docking, CH_3_Cd^+^ and CH_3_CdOH bind in the same region, with Se···Cd distances of 3.4–3.5 Å, while CH_3_CdCys interacts with the Sec498, Asn419, and Gln494.

In general, in TrxR1, shorter Se···Cd distances (3.4–5.2 Å) are observed compared to those of GPx1 (3.9–6.3 Å). Due to the relatively short distances between the Sec residue of selenoenzymes and the metal center, a nucleophilic attack from Se to Cd might indeed occur, forming the Se–Cd covalent bond and consequently leading to enzyme inhibition. A reasonable mechanistic hypothesis suggests that the CH_3_Cd^+^ moiety can be transferred from CH_3_CdCys to the selenoenzymes, through an exchange reaction similar to the Rabenstein reactions.^61,111,112^

## 4 Conclusions

The toxicity of Hg^2+^, CH_3_Hg^+^, and Cd^2+^ is largely governed by their interactions with abundant thiol-containing biomolecules, but their affinity for the comparatively scarce selenoproteins also plays an important role. In contrast, the available data on organic Cd species, such as methylcadmium, is still considerably limited. In this work, the effects of CH_3_Cd^+^ binding on the H_2_O_2_ reducing potential of Cys and Sec residues have been studied with a scalar relativistic DFT approach at the ZORA-BLYP-D3­(BJ)/TZ2P level of theory. The reactions of the neutral amino acids and their toxified analogues with H_2_O_2_ have been modeled both in the gas phase and in solvent via implicit solvation proton transfer and SAPE mechanisms.

CH_3_Cd^+^ substitution facilitates the oxidation by H_2_O_2_ compared to neutral Cys and Sec; on the other hand, the energy barrier for the reaction is much higher when compared to that of the deprotonated residues (Cys^–^ and Sec^–^). CH_3_CdCys and CH_3_CdSec display a more destabilized HOMO, which lowers the activation energy for the reduction of hydroperoxides, in line with previous findings involving methylmercury binding.^60^ However, the release of methylcadmium hydroxide by cleavage of the Cd–chalcogen bond (not observed for CH_3_Hg^+^-substituted amino acids) suggests that the metal might consecutively target multiple (seleno)­cysteines in a residue-hopping mechanism and promote their oxidation, with severe effects on the cell’s redox equilibrium. Both minimal and SAPE energy barriers for the reduction of H_2_O_2_ by CH_3_Cd^+^-substituted Cys and Sec are higher compared to those reported for the analogous CH_3_Hg^+^-substituted amino acids,^60^ which is likely ascribed to the less diffuse character of Cd 4d outer shell orbitals and matches the trends in the affinity for –SH and –SeH groups. This periodic trend (Cd-4d vs Hg-5d) is of chemical and toxicological interest and needs to be confirmed in biochemical models. The favorable binding by the metal to the catalytic pockets of GPx1 and TrxR1 hints at the occurrence of Rabenstein-like CH_3_Cd^+^ transfer reactions from CH_3_CdCys to Sec, resulting in a loss of enzymatic function. The pro-oxidant toxicity of CH_3_Cd^+^ is deemed comparable to that of methylmercury. The detailed insight into methylcadmium chalcogenolate chemistry provided by this work prompts further experimental investigation, which will help expand our limited knowledge on the topic and derive efficient protocols for detoxification.

## Supplementary Material





## Notes

Supplementary data and figures, including gas-phase Gibbs energies, single-point electronic energies in solvent, ASA strain energies, EDA dispersion energies, NOCV deformation densities, Cartesian coordinates, ADF energies, and imaginary frequencies (PDF)
All optimized Cartesian coordinates, provided as a separate file (XYZ)
