# Supplementary material for: Molecular Aspects of Methylcadmium Toxicity: Effects on the H2O2 Reduction by Cysteine and Selenocysteine Disclosed In Silico
Source: Chem Res Toxicol. 2025 Dec 11;39(1):144–56. doi: 10.1021/acs.chemrestox.5c00409 (PMC12820964; doi:10.1021/acs.chemrestox.5c00409)
Supplement: Supplementary file 1 [file tx5c00409_si_002.pdf]

Supporting Information

Molecular Aspects of Methylcadmium Toxicity: Effects on the H_2O_2 Reduction by Cysteine and Selenocysteine Disclosed in Silico

Alessandro Rubbi,[1] Francesco Lambertini,[1] Pablo Andrei Nogara,[2,3] Marco Bortoli,[4] João B. T. Rocha,[3] Laura Orian[1]*

[1] Dipartimento di Scienze Chimiche, Università degli Studi di Padova, via Marzolo 1, 35131 Padova, Italy.
[2] Federal Institute of Education, Science and Technology Sul-rio-grandense (IFSul), Av. Leonel de Moura Brizola, 2501, 96418-400 Bagé, RS, Brazil.
[3] Department of Biochemistry and Molecular Biology, Center for Natural and Exact Sciences, Federal University of Santa Maria, Camobi, Santa Maria 97105-900, Brazil.
[4] Department of Chemistry and Hylleraas Centre for Quantum Molecular Sciences, University of Oslo, PO Box 1033, 0315 Oslo, Norway.
* laura.orian@unipd.it

Table of Contents

Table S1. Gas-phase Gibbs free energies (298 K, 1 atm) relative to free reactants of stationary points for the oxidation of **Cys**, **Sec**, **CH_3CdCys**, **CH_3CdSec**, **Cys$^-$** and **Sec$^-$** by H_2O_2 (*Table 1*, *Figure 1* in the main text)……S2

Table S2. Gas-phase Gibbs free energies (298 K, 1 atm) relative to free reactants of SAPE stationary points for the oxidation of **Cys**, **Sec**, **CH_3CdCys** and **CH_3CdSec** by H_2O_2 (*Table 2*, *Figure 2* in the main text)……S2

Table S3. Single-point electronic energies in H_2O relative to free reactants of stationary points for the oxidation of **Cys**, **Sec**, **CH_3CdCys**, **CH_3CdSec**, **Cys$^-$** and **Sec$^-$** by H_2O_2 (*Table 1*, *Figure 1* in the main text)……S2

Table S4. Single-point electronic energies in H_2O relative to free reactants of SAPE stationary points for the oxidation of **Cys**, **Sec**, **CH_3CdCys** and **CH_3CdSec** by H_2O_2 (*Table 2*, *Figure 2* in the main text)……S2

Table S5. Strain energies at the TS from ASA of **Cys**, **Sec**, **CH_3CdCys** and **CH_3CdSec** (*Figure 3* in the main text)……S3

Table S6. Interaction energies and dispersion contributions at $d_{Ch\text{-}O}$~2.15 Å from EDA of **Cys**, **Sec**, **CH_3Cd** and **CH_3CdSec** (*Figure 3* in the main text)……S3

Figure S1. Major NOCV deformation densities of **Cys**, **Sec**, **CH_3CdCys** and **CH_3CdSec**, associated with the HOMO-LUMO charge transfer between the interacting fragments ($d_{Ch\text{-}O}$~2.15 Å) (Table 3, Figure 4 in the main text)……S4

Table S7. Cartesian Coordinates (in Å), ADF electronic energies (in Hartree) and imaginary frequencies (Nimag)……S5

Table S1. Gas-phase Gibbs free energies (298 K, 1 atm) relative to free reactants of stationary points for the oxidation of **Cys**, **Sec**, **CH_3CdCys**, **CH_3CdSec**, **Cys^-** and **Sec^-** by H_2O_2 (*Table 1*, *Figure 1* in the main text).

	RC †	TS	PC	TS_{iso}	P
**Cys**	3.6	26.0 (26.0)	-36.3	-0.2	-48.2
**Sec**	3.5	22.1 (22.1)	-29.3	-0.01	-53.4
**CH_3CdCys**	1.9	23.0 (23.0)	-36.9		-32.1
**CH_3CdSec**	0.3	19.8 (19.8)	-30.9		-23.4
**Cys^-**	-9.9	-3.0 (6.9)	-55.0		-49.8
**Sec^-**	-9.1	-4.0 (5.1)	-50.3		-44.2

Activation energies relative to the RC are given in parentheses. All energies are in in kcal·mol^{-1}. Level of theory: ZORA-BLYP-D3(BJ)/TZ2P.

Table S2. Gas-phase Gibbs free energies (298 K, 1 atm) relative to free reactants of SAPE stationary points for the oxidation of **Cys**, **Sec**, **CH_3CdCys** and **CH_3CdSec** by H_2O_2 (*Table 2*, *Figure 2* in the main text).

	RC †	TS	PC
**Cys**	7.3	20.2	-45.5
**Sec**	10.8	17.5	-49.5
**CH_3CdCys**	6.2	13.1	-37.4
**CH_3CdSec**	7.0	11.2	-35.6

All energies are in in kcal·mol^{-1}. Level of theory: ZORA-BLYP-D3(BJ)/TZ2P.

Table S3. Single-point electronic energies in H_2O relative to free reactants of stationary points for the oxidation of **Cys**, **Sec**, **CH_3CdCys**, **CH_3CdSec**, **Cys^-** and **Sec^-** by H_2O_2 (*Table 1*, *Figure 1* in the main text).

	RC	TS	PC	TS_{iso}	P
**Cys**	-4.0	6.7 (10.7)	-47.9	-3.7	-52.3
**Sec**	-3.9	2.0 (5.9)	-41.3	-5.1	-57.9
**CH_3CdCys**	-7.2	1.3 (8.5)	-50.1		-40.2
**CH_3CdSec**	-7.6	-3.1 (4.5)	-45.5		-34.1
**Cys^-**	-8.5	-3.4 (5.1)	-58.7		-49.9
**Sec^-**	-8.0	-2.0 (6.0) [a]	-55.5		-45.9

Activation energies relative to the RC are given in parentheses. All energies are in in kcal·mol^{-1}. Level of theory: COSMO-ZORA-BLYP-D3(BJ)/TZ2P//ZORA-BLYP-D3(BJ)/TZ2P. [a] In this case, single-point calculations fail to assign the most negative TS energy to **Sec^-**. Geometry re-optimization in solvent would this inconsistency, but this falls outside the scope of the work.

Table S4. Single-point electronic energies in H_2O relative to free reactants of SAPE stationary points for the oxidation of **Cys**, **Sec**, **CH_3CdCys** and **CH_3CdSec** by H_2O_2 (*Table 2*, *Figure 2* in the main text).

	RC	TS	PC
**Cys**	-14.1	-6.1 (8.0)	-71.2
**Sec**	-10.9	-5.9 (5.0)	-75.9
**CH_3CdCys**	-17.6	-12.4 (5.2)	-60.6
**CH_3CdSec**	-16.7	-12.7 (4.0)	-60.1

Activation energies relative to the RC are given in parentheses. All energies are in in kcal·mol^{-1}. Level of theory: COSMO-ZORA-BLYP-D3(BJ)/TZ2P//ZORA-BLYP-D3(BJ)/TZ2P.

† Neutral RCs are more stable compared to the free reactants in terms of electronic energy (Table 1, main text) but are destabilized in terms of Gibbs free energy (Tables S1 and S2). As a result, energy barrier trends in ΔE ΔG do not correspond completely. However, TS energies follow the same trends in electronic and Gibbs free energies, allowing for a comparison of the studied reactions. The same observations are valid for COSMO energies (see Tables S3-S4).

Table S5. Strain energies at the TS from ASA of **Cys**, **Sec**, **CH_3CdCys** and **CH_3CdSec** (*Figure 3* in the main text).

	ΔE_{strain} [a]	$\Delta E_{strain,chalc.}$ [b]	$\Delta E_{strain,H_2O_2}$ [c]	$d_{O\text{-}O}$ [d]
**Cys**	43.6	0.8	42.7	2.05
**Sec**	44.0	0.8	43.2	2.06
**CH_3CdCys**	40.3	0.7	39.7	2.04
**CH_3CdSec**	40.5	0.7	39.9	2.05

[a] Strain energy from ASA. [b,c] Fragment strain energies (chalc. = aminoacidic fragment). [d] O-O interatomic distance in the H_2O_2 fragment, in Å. All energies are in in kcal·mol^{-1}. Level of theory: ZORA-BLYP-D3(BJ)/TZ2P.

Table S6. Interaction energies and dispersion contributions at $d_{Ch\text{-}O}$~2.15 Å from EDA of **Cys**, **Sec**, **CH_3Cd** and **CH_3CdSec** (*Figure 3* in the main text).

	ΔE_{int}	ΔE_{disp}
**Cys**	-10.1	-2.5
**Sec**	-23.6	-2.7
**CH_3CdCys**	-15.3	-3.4
**CH_3CdSec**	-29.6	-3.3

All energies are in in kcal·mol^{-1}. Level of theory: ZORA-BLYP-D3(BJ)/TZ2P.

Figure S1. Major NOCV deformation densities of **Cys**, **Sec**, **CH_3CdCys** and **CH_3CdSec**, associated with the HOMO-LUMO charge transfer between the interacting fragments ($d_{Ch\text{-}O}$~2.15 Å) (*Table 3*, *Figure 4* in the main text). Blue and red regions represent accumulation and depletion of electronic density, respectively ($\Delta\rho$ > 0.005). Level of theory: ZORA-BLYP-D3(BJ)/TZ2P.

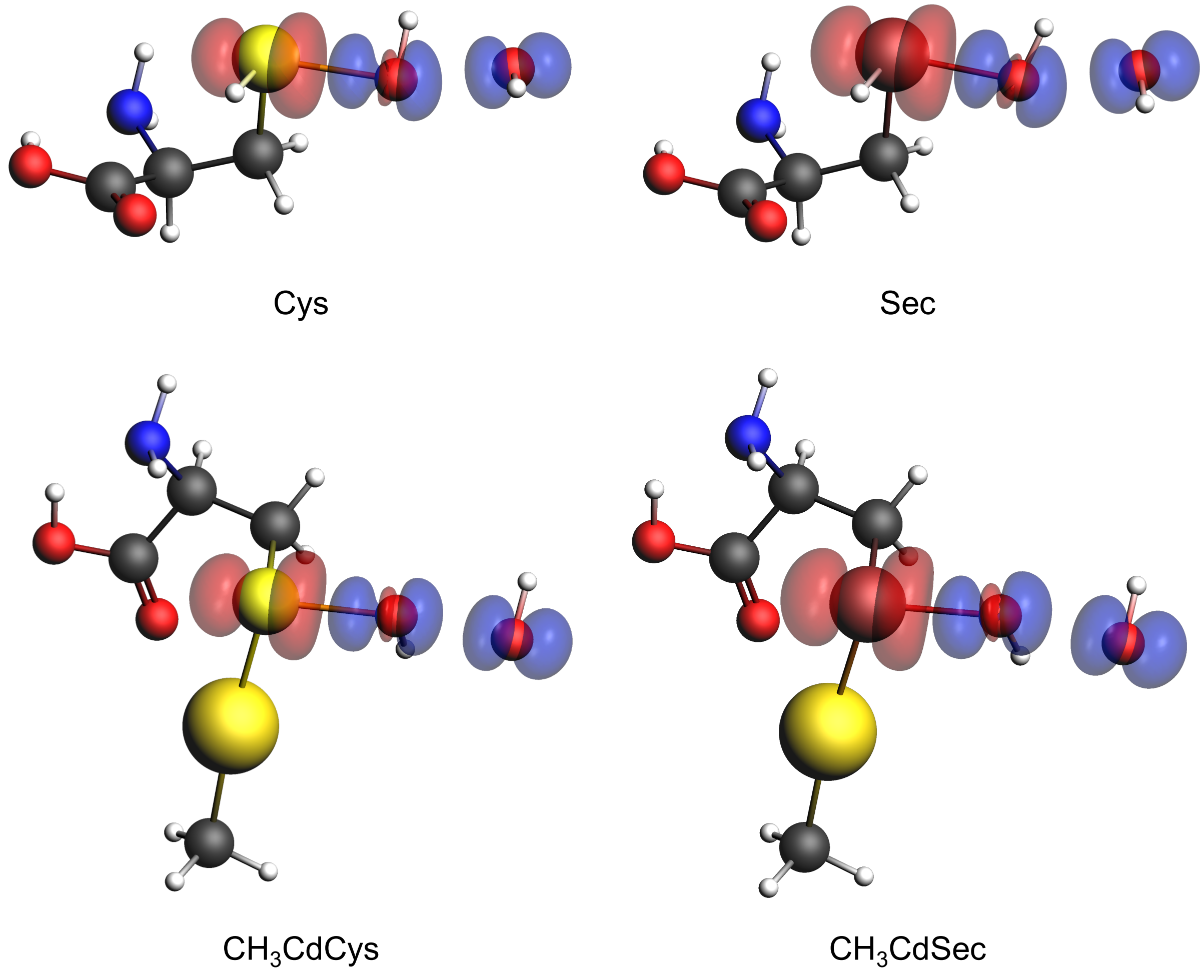

Table S7. Cartesian Coordinates (in Å), ADF electronic energies (in Hartree) and imaginary frequencies (Nimag).

H_2O
-0.50675052 Nimag=0

O	0.000000000	0.000000000	2.549926000
H	0.000000000	0.768789000	1.954184000
H	0.000000000	-0.768789000	1.954184000

H_2O_2
-0.64253209 Nimag=0

O	-0.727403000	0.173325000	0.297172000
H	-0.697497000	0.990631000	-0.239891000
H	0.697497000	-0.990631000	-0.239891000
O	0.727403000	-0.173325000	0.297172000

Cys
-2.77711318 Nimag=0

C	1.336987000	-3.312413000	7.397812000
C	2.655103000	-3.484665000	6.620510000
N	1.473040000	-2.906188000	8.811380000
H	2.319801000	-3.326721000	9.206056000
H	1.541958000	-1.894694000	8.909987000
H	0.742871000	-2.543709000	6.882541000
C	0.476439000	-4.607301000	7.312175000
O	0.410982000	-5.296515000	6.315773000
O	-0.212661000	-4.870886000	8.445941000
H	0.084470000	-4.162157000	9.082878000
H	2.450388000	-3.831563000	5.606032000
H	3.188660000	-2.530493000	6.570421000
S	3.839338000	-4.651185000	7.431309000
H	3.129163000	-5.773162000	7.165610000

Cys^-
-2.68869302 Nimag=0

C	1.276547000	-3.125803000	7.449937000
C	2.636044000	-3.280113000	6.695914000
N	1.440697000	-3.028617000	8.926590000
H	2.385390000	-3.455280000	9.095219000
H	1.479487000	-2.050474000	9.215854000
H	0.768057000	-2.221042000	7.081603000
C	0.291236000	-4.280907000	7.142478000
O	-0.040907000	-4.619922000	6.019227000
O	-0.236774000	-4.839495000	8.263098000
H	0.231462000	-4.308385000	8.988367000
H	2.417465000	-3.702550000	5.709125000
H	3.046190000	-2.268491000	6.543589000
S	3.847177000	-4.325182000	7.619898000

Sec
-2.74303072 Nimag=0

C	1.327459000	-3.297539000	7.396030000
C	2.635075000	-3.446546000	6.605687000
N	1.464251000	-2.895199000	8.810821000
H	2.316050000	-3.309627000	9.202288000
H	1.522956000	-1.883438000	8.913272000
H	0.720567000	-2.531466000	6.890151000
C	0.473951000	-4.597353000	7.305832000
O	0.399068000	-5.274078000	6.301942000
O	-0.200974000	-4.876733000	8.444438000
H	0.095877000	-4.170538000	9.084277000
H	2.438115000	-3.815438000	5.599103000
H	3.168295000	-2.493559000	6.552626000
Se	3.946620000	-4.698030000	7.466539000
H	3.129229000	-5.902109000	7.185416000

Sec^-
-2.66591724 Nimag=0

C	1.320279000	-3.298299000	7.377777000
C	2.669233000	-3.484821000	6.640396000
N	1.460485000	-2.966052000	8.823499000
H	2.404351000	-3.333885000	9.086316000
H	1.473478000	-1.953738000	8.954167000
H	0.757124000	-2.485974000	6.889089000
C	0.391405000	-4.534083000	7.246671000
O	0.103536000	-5.066592000	6.189103000
O	-0.151331000	-4.916291000	8.432171000
H	0.279625000	-4.261088000	9.071894000
H	2.489129000	-4.047197000	5.721303000
H	3.067174000	-2.496735000	6.377019000
Se	4.042816000	-4.444784000	7.763578000

CH_3CdCys
-3.37230825 Nimag=0

C	-0.611405000	0.754797000	-0.120347000
C	0.784365000	0.609165000	-0.748522000
N	-0.632726000	1.228987000	1.281151000
H	0.188030000	0.846468000	1.764083000
H	-0.586371000	2.245518000	1.331946000
H	-1.191439000	1.468425000	-0.725615000
C	-1.407489000	-0.573838000	-0.191399000
O	-1.311103000	-1.375281000	-1.109086000
O	-2.230540000	-0.763617000	0.854522000
H	-2.015921000	0.005087000	1.463555000
H	0.688073000	0.265615000	-1.780338000
H	1.277591000	1.586480000	-0.757070000
S	1.899499000	-0.522972000	0.217941000
Cd	1.073765000	-2.678225000	-0.548119000
C	0.574891000	-4.669059000	-1.171009000
H	0.960501000	-4.838993000	-2.179975000
H	1.009892000	-5.398315000	-0.482678000
H	-0.514758000	-4.753010000	-1.170239000

CH_3CdSec
-3.34761612 Nimag=0

C	-0.612374000	0.766452000	-0.129735000
C	0.786722000	0.661876000	-0.746058000
N	-0.668785000	1.259603000	1.264269000
H	0.149731000	0.901481000	1.769907000
H	-0.646130000	2.277545000	1.301632000
H	-1.210662000	1.454478000	-0.748352000
C	-1.371503000	-0.584266000	-0.195023000
O	-1.245104000	-1.391725000	-1.103636000
O	-2.203586000	-0.782994000	0.841780000
H	-2.019123000	-0.001541000	1.444172000
H	0.726812000	0.283578000	-1.766480000
H	1.265292000	1.644462000	-0.756969000
Se	2.027617000	-0.519005000	0.323320000
Cd	1.098278000	-2.736984000	-0.518453000
C	0.543731000	-4.707564000	-1.178074000
H	0.736501000	-4.788846000	-2.251367000
H	1.121293000	-5.463119000	-0.639429000
H	-0.523855000	-4.836198000	-0.982703000

Cys-RC
-3.42922462 Nimag=0

C	1.348653000	-3.315367000	7.319494000
C	2.744921000	-3.611128000	6.750733000
N	1.312641000	-2.605195000	8.614542000

H	2.079728000	-2.930042000	9.209418000
H	1.409835000	-1.598178000	8.494465000
H	0.820040000	-2.690750000	6.584464000
C	0.501794000	-4.617188000	7.422165000
O	0.583007000	-5.527972000	6.624787000
O	-0.353456000	-4.621626000	8.470540000
H	-0.146969000	-3.778769000	8.961750000
H	2.667461000	-4.136555000	5.797761000
H	3.308838000	-2.686991000	6.601332000
S	3.795039000	-4.620997000	7.895768000
H	3.160947000	-5.795987000	7.668762000
O	6.049188000	-3.915517000	5.642778000
O	5.887980000	-2.512684000	6.133856000
H	6.788862000	-2.356802000	6.480224000
H	5.500220000	-4.383312000	6.322291000

Cys$^-$-RC
-3.36287681 Nimag=0

C	1.419857000	-3.047497000	7.538900000
C	2.719883000	-3.244349000	6.693735000
N	1.634294000	-3.057977000	9.007539000
H	2.462292000	-3.677485000	9.132635000
H	1.958597000	-2.137431000	9.311666000
H	0.970035000	-2.082890000	7.258648000
C	0.323567000	-4.091751000	7.204569000
O	-0.016953000	-4.398913000	6.075557000
O	-0.282044000	-4.586354000	8.315465000
H	0.233178000	-4.113791000	9.051407000
H	2.429090000	-3.652547000	5.720870000
H	3.164208000	-2.253410000	6.516797000
S	3.965766000	-4.338703000	7.508758000
O	3.997994000	-0.925669000	9.108478000
O	5.156941000	-1.882238000	8.960643000
H	4.717746000	-2.723142000	8.534914000
H	3.993810000	-0.540986000	8.210743000

Sec-RC
-3.39516085 Nimag=0

C	1.460242000	-3.393093000	7.123020000
C	2.760064000	-3.917538000	6.505247000
N	1.597069000	-2.599041000	8.361781000
H	2.345143000	-2.988602000	8.942469000
H	1.824451000	-1.626192000	8.162303000
H	0.980420000	-2.747358000	6.371696000
C	0.441592000	-4.546874000	7.357790000
O	0.334463000	-5.500487000	6.616073000
O	-0.334506000	-4.364554000	8.451312000
H	0.020588000	-3.532338000	8.870547000
H	2.559618000	-4.516305000	5.617304000
H	3.443911000	-3.104306000	6.254381000
Se	3.821803000	-5.068242000	7.765752000
H	5.388166000	-5.117467000	5.878348000
O	5.993370000	-3.307929000	5.666410000
H	6.945505000	-3.279845000	5.886736000
O	5.901162000	-4.703400000	5.138382000
H	2.907688000	-6.227702000	7.635493000

Sec$^-$-RC
-3.33825607 Nimag=0

C	1.447808000	-3.060352000	7.523790000
C	2.696932000	-3.302679000	6.637482000
N	1.707493000	-2.988809000	8.984388000
H	2.509367000	-3.633151000	9.138215000
H	2.076146000	-2.064837000	9.221117000
H	0.987830000	-2.107007000	7.217177000
C	0.327582000	-4.107832000	7.284967000
O	-0.040874000	-4.488318000	6.188173000
O	-0.268446000	-4.504027000	8.439480000
H	0.272385000	-3.996704000	9.131268000
H	2.378938000	-3.743860000	5.690487000
H	3.181169000	-2.341819000	6.427113000
Se	4.067277000	-4.504584000	7.496217000
O	4.070403000	-0.872188000	8.986712000
O	5.243982000	-1.820349000	8.895741000
H	4.822305000	-2.682803000	8.519720000
H	4.074184000	-0.526905000	8.073062000

CH_3CdCys-RC
-4.03339212 Nimag=0

C	1.783400000	-3.166161000	7.319448000
C	3.320052000	-3.183700000	7.314572000
N	1.152359000	-3.338412000	8.646847000
H	1.797051000	-3.858510000	9.252517000
H	0.947185000	-2.443770000	9.087737000
H	1.453709000	-2.205615000	6.897701000
C	1.208641000	-4.243324000	6.360203000
O	1.669427000	-4.487172000	5.254087000
O	0.141110000	-4.886748000	6.857668000
H	0.047869000	-4.490360000	7.781069000
H	3.681043000	-3.153391000	6.285208000
H	3.693541000	-2.297016000	7.836385000
S	4.077420000	-4.622281000	8.222440000
Cd	3.215871000	-6.673589000	7.155994000
C	2.466137000	-8.611799000	6.619496000
H	1.568510000	-8.464149000	6.013171000
H	3.229220000	-9.130292000	6.033576000
H	2.221643000	-9.188252000	7.514696000
H	5.263982000	-4.771763000	6.147404000
O	4.168753000	-5.709857000	4.861009000
H	3.412207000	-5.086426000	4.730727000
O	5.285358000	-4.766261000	5.153460000

CH_3CdSec-RC
-4.00913965 Nimag=0

C	1.771677000	-3.141349000	7.307787000
C	3.303796000	-3.130463000	7.321495000
N	1.117899000	-3.257707000	8.630398000
H	1.745530000	-3.760974000	9.267881000
H	0.912431000	-2.345317000	9.033246000
H	1.431406000	-2.205589000	6.838771000
C	1.220101000	-4.265227000	6.389992000
O	1.699085000	-4.555224000	5.302696000
O	0.146767000	-4.890180000	6.896224000
H	0.033772000	-4.453040000	7.798598000
H	3.694670000	-3.152242000	6.304740000
H	3.667821000	-2.231723000	7.825762000
Se	4.136744000	-4.642599000	8.374412000
Cd	3.238114000	-6.744852000	7.175006000
C	2.463872000	-8.656039000	6.560034000
H	1.555459000	-8.473718000	5.979761000
H	3.212506000	-9.149425000	5.935147000
H	2.233660000	-9.272206000	7.432312000
H	5.307591000	-4.797522000	6.151464000
O	4.193855000	-5.743229000	4.886221000
H	3.422391000	-5.134080000	4.775150000
O	5.295351000	-4.776295000	5.158205000

Cys-TS
-3.39122151 Nimag=1 v=-605.3

C	1.252789000	-3.335821000	7.332923000

C	2.619481000	-3.546193000	6.663427000
N	1.238573000	-2.551459000	8.583267000
H	2.029762000	-2.798740000	9.181425000
H	1.279940000	-1.549429000	8.403676000
H	0.625929000	-2.797739000	6.606723000
C	0.520543000	-4.690077000	7.563544000
O	0.678701000	-5.658605000	6.850310000
O	-0.325436000	-4.672786000	8.617111000
H	-0.184788000	-3.785132000	9.044407000
H	2.524466000	-4.050201000	5.700244000
H	3.138018000	-2.595335000	6.516581000
S	3.785853000	-4.554230000	7.669073000
H	6.067233000	-4.457161000	6.542359000
O	6.889293000	-4.369353000	5.149133000
H	6.860086000	-5.276915000	4.789574000
O	5.139646000	-4.578685000	6.188957000
H	3.182750000	-5.742172000	7.414343000

Cys^--TS

-3.35195360 Nimag=1 v=-218.1

C	1.282929000	-3.135948000	7.450384000
C	2.634804000	-3.273189000	6.688523000
N	1.429283000	-3.018454000	8.924527000
H	2.325965000	-3.501027000	9.134904000
H	1.547039000	-2.041770000	9.195148000
H	0.765840000	-2.240146000	7.075598000
C	0.307179000	-4.302218000	7.146286000
O	0.033339000	-4.692018000	6.025826000
O	-0.274883000	-4.811984000	8.263773000
H	0.156015000	-4.259375000	8.991578000
H	2.425333000	-3.701084000	5.703185000
H	3.060325000	-2.272141000	6.555775000
S	3.887029000	-4.293693000	7.576500000
O	5.164181000	-0.612873000	9.428554000
O	5.013897000	-2.005933000	8.429772000
H	4.486706000	-2.572531000	9.028818000
H	4.704078000	-0.040749000	8.787775000

Sec-TS

-3.36315446 Nimag=1 v=-548.2

C	1.245367000	-3.334059000	7.319797000
C	2.614903000	-3.583863000	6.685918000
N	1.219845000	-2.521757000	8.553193000
H	1.998520000	-2.765235000	9.169417000
H	1.277493000	-1.524574000	8.352276000
H	0.645822000	-2.796486000	6.569342000
C	0.470858000	-4.664398000	7.556749000
O	0.596454000	-5.639469000	6.846959000
O	-0.375047000	-4.614630000	8.610267000
H	-0.214525000	-3.724123000	9.025072000
H	2.543623000	-4.146557000	5.755243000
H	3.164433000	-2.656472000	6.513554000
Se	3.859310000	-4.650013000	7.833493000
H	6.176013000	-4.605881000	6.535521000
O	6.946891000	-4.277281000	5.139454000
H	6.761795000	-5.054104000	4.577117000
O	5.225826000	-4.517093000	6.238855000
H	3.165256000	-5.934039000	7.534850000

Sec^--TS

-3.33031723 Nimag=1 v=-254.5

C	1.483858000	-3.082964000	7.477091000
C	2.712311000	-3.344163000	6.568283000
N	1.745146000	-2.999851000	8.935628000
H	2.485736000	-3.698344000	9.118819000
H	2.162747000	-2.093905000	9.175069000
H	1.042688000	-2.122023000	7.168781000
C	0.337867000	-4.106748000	7.253303000
O	-0.022966000	-4.515456000	6.164741000
O	-0.286544000	-4.451316000	8.410517000
H	0.255572000	-3.939484000	9.095925000
H	2.371819000	-3.792796000	5.632568000
H	3.217968000	-2.398892000	6.364599000
Se	4.124248000	-4.525406000	7.372546000
O	4.150417000	-0.747411000	9.512268000
O	4.782580000	-2.021309000	8.479581000
H	4.358741000	-2.782998000	8.926616000
H	4.026871000	-0.152067000	8.750591000

CH_3CdCys-TS

-3.99231302 Nimag=1 v=-485.7

C	1.614005000	-4.339536000	6.135046000
C	3.140057000	-4.419372000	6.287223000
N	0.995797000	-3.028780000	6.428721000
H	1.491004000	-2.571488000	7.198853000
H	1.029647000	-2.408423000	5.621420000
H	1.370542000	-4.592512000	5.092083000
C	0.892420000	-5.420454000	6.982891000
O	1.366151000	-6.523435000	7.219870000
O	-0.315881000	-5.041959000	7.422777000
H	-0.382602000	-4.079992000	7.147468000
H	3.503454000	-5.391904000	5.950421000
H	3.621573000	-3.643399000	5.685698000
S	3.705926000	-4.107915000	8.024364000
Cd	3.379927000	-6.366554000	8.990325000
C	3.340701000	-8.260135000	9.989175000
H	3.167824000	-8.099378000	11.055780000
H	2.525995000	-8.842620000	9.553472000
H	4.298116000	-8.763149000	9.835950000
H	6.009482000	-4.985341000	7.395887000
O	7.615223000	-4.570737000	7.032862000
H	7.574523000	-3.992530000	6.247931000
O	5.727734000	-4.062779000	7.608434000

CH_3CdSec-TS

-3.97272703 Nimag=1 v=-436.7

C	1.600487000	-4.343104000	6.131778000
C	3.123040000	-4.380743000	6.278236000
N	0.947614000	-3.039721000	6.384960000
H	1.429910000	-2.544133000	7.139598000
H	0.964167000	-2.444594000	5.558320000
H	1.358645000	-4.632587000	5.097033000
C	0.902340000	-5.417165000	7.007758000
O	1.395482000	-6.506860000	7.265288000
O	-0.311027000	-5.050946000	7.444161000
H	-0.398708000	-4.098409000	7.142709000
H	3.521972000	-5.354521000	5.995330000
H	3.595634000	-3.609800000	5.666127000
Se	3.756972000	-3.976520000	8.149390000
Cd	3.373839000	-6.375718000	9.063434000
C	3.298163000	-8.313769000	9.985216000
H	3.122198000	-8.195025000	11.056842000
H	2.475333000	-8.861260000	9.520315000
H	4.247249000	-8.827066000	9.814883000
H	6.135233000	-4.920759000	7.535533000
O	7.711399000	-4.528780000	6.980453000
H	7.590050000	-4.107511000	6.108656000
O	5.831625000	-3.983402000	7.580632000

Cys-PC

-3.49594477 Nimag=0

C	1.525751000	-3.459099000	6.999857000
C	1.968228000	-4.423289000	5.884888000
N	2.474016000	-2.447120000	7.488385000
H	3.442683000	-2.782343000	7.593375000
H	2.491531000	-1.622917000	6.891281000
H	0.654021000	-2.917255000	6.604450000
C	0.959202000	-4.263360000	8.210865000
O	0.400166000	-5.335683000	8.094689000
O	1.127109000	-3.626702000	9.387280000
H	1.667646000	-2.817978000	9.144578000
H	1.115540000	-5.027432000	5.563466000
H	2.408975000	-3.898705000	5.031346000
S	3.285654000	-5.634474000	6.451396000
H	5.305058000	-3.948349000	7.004954000
O	5.337835000	-3.184527000	7.636163000
H	5.866965000	-3.486427000	8.392765000
O	4.629758000	-5.175989000	5.893722000
H	2.824359000	-6.596549000	5.567741000

Cys$^-$-PC
-3.43503588 Nimag=0

C	1.355022000	-3.035892000	7.306612000
C	2.610437000	-3.258271000	6.441452000
N	1.539536000	-2.310690000	8.586870000
H	2.495925000	-2.378151000	8.974964000
H	1.342464000	-1.318261000	8.472555000
H	0.621632000	-2.466290000	6.707346000
C	0.621641000	-4.376801000	7.605082000
O	0.379068000	-5.227258000	6.766011000
O	0.205264000	-4.464630000	8.890265000
H	0.571543000	-3.598334000	9.277453000
H	2.332320000	-3.893051000	5.591181000
H	2.989531000	-2.298433000	6.065687000
S	4.052896000	-4.062610000	7.294931000
O	4.138499000	-1.951318000	10.012356000
O	4.979019000	-2.824087000	7.805920000
H	4.550635000	-2.343547000	9.095264000
H	4.340026000	-1.002511000	9.960276000

Sec-PC
-3.44936926 Nimag=0

C	1.479554000	-3.506824000	6.952843000
C	2.077266000	-4.530871000	5.979535000
N	2.313464000	-2.367089000	7.370546000
H	3.310972000	-2.606463000	7.482513000
H	2.255204000	-1.598342000	6.705497000
H	0.585941000	-3.091929000	6.462465000
C	0.920552000	-4.221266000	8.221760000
O	0.486963000	-5.357730000	8.208706000
O	0.957291000	-3.441649000	9.318162000
H	1.449945000	-2.623469000	9.004774000
H	1.303552000	-5.183929000	5.572942000
H	2.662204000	-4.068652000	5.180884000
Se	3.422183000	-5.783742000	6.888324000
H	5.282176000	-3.846665000	6.974856000
O	5.196222000	-2.968236000	7.444928000
H	5.821955000	-2.999782000	8.187012000
O	4.916656000	-5.359709000	6.202828000
H	2.968499000	-6.935555000	6.002223000

Sec$^-$-PC
-3.40393794 Nimag=0

C	1.331646000	-3.025068000	7.321550000
C	2.590818000	-3.225767000	6.469791000
N	1.508225000	-2.337948000	8.624585000
H	2.483127000	-2.326905000	8.983393000
H	1.210992000	-1.366211000	8.563457000
H	0.605683000	-2.438782000	6.729600000
C	0.595428000	-4.375747000	7.574706000
O	0.342193000	-5.190228000	6.702684000
O	0.200701000	-4.513037000	8.859717000
H	0.585402000	-3.662697000	9.273072000
H	2.326183000	-3.756318000	5.549958000
H	3.061020000	-2.266819000	6.227339000
Se	4.080982000	-4.269522000	7.369703000
O	4.108616000	-1.844960000	9.892853000
O	5.161739000	-2.949131000	7.913176000
H	4.611892000	-2.352470000	9.053697000
H	4.320813000	-0.908525000	9.744944000

CH_3CdCys-PC
-4.09330227 Nimag=0

C	1.440964000	-3.195434000	7.230352000
C	2.870492000	-3.335440000	6.693589000
N	1.316346000	-2.609752000	8.586076000
H	2.125296000	-2.869571000	9.156144000
H	1.273623000	-1.592725000	8.551116000
H	0.881846000	-2.550078000	6.538004000
C	0.671916000	-4.545735000	7.198213000
O	0.829601000	-5.388510000	6.329882000
O	-0.206542000	-4.689463000	8.205862000
H	-0.048081000	-3.883241000	8.779317000
H	2.874381000	-3.674966000	5.655613000
H	3.399161000	-2.378808000	6.758576000
S	3.901301000	-4.500797000	7.747955000
Cd	3.277235000	-6.679516000	6.493659000
C	2.752194000	-8.668374000	5.833341000
H	2.594723000	-8.629131000	4.752103000
H	3.562454000	-9.363124000	6.068648000
H	1.831970000	-8.976366000	6.334315000
H	5.433478000	-5.050437000	5.892075000
O	5.134553000	-5.650346000	5.113741000
H	5.883027000	-6.235573000	4.911606000
O	5.379364000	-4.250014000	7.274414000

CH_3CdSec-PC
-4.05791370 Nimag=0

C	1.456815000	-3.170439000	7.220613000
C	2.917635000	-3.265289000	6.784791000
N	1.219256000	-2.608897000	8.573713000
H	2.008779000	-2.811584000	9.191035000
H	1.105065000	-1.597350000	8.543632000
H	0.928473000	-2.526839000	6.501328000
C	0.728342000	-4.539529000	7.110708000
O	0.948806000	-5.347755000	6.223861000
O	-0.185806000	-4.743552000	8.075560000
H	-0.085001000	-3.945923000	8.673927000
H	3.011429000	-3.621837000	5.758784000
H	3.428966000	-2.305322000	6.890510000
Se	4.028731000	-4.540092000	7.937634000
Cd	3.331672000	-6.726473000	6.473763000
C	2.699721000	-8.702000000	5.852913000
H	2.504537000	-8.665816000	4.777854000
H	3.488346000	-9.427669000	6.067172000
H	1.786462000	-8.965562000	6.390395000
H	5.434798000	-5.054484000	5.912768000
O	5.052470000	-5.635325000	5.118355000
H	5.772027000	-6.213642000	4.816104000
O	5.597776000	-4.302022000	7.209183000

Cys-TS$_{iso}$

-2.91136481 Nimag=1 v=-1462.7

C	1.251608000	-3.388310000	7.211355000
C	2.402158000	-3.919324000	6.338710000
N	1.667926000	-2.689898000	8.448597000
H	2.620323000	-2.959360000	8.721050000
H	1.631983000	-1.678286000	8.346096000
H	0.664169000	-2.698621000	6.589948000
C	0.262763000	-4.546320000	7.563044000
O	-0.112121000	-5.359307000	6.743060000
O	-0.140111000	-4.524337000	8.848120000
H	0.377573000	-3.760426000	9.243770000
H	1.999681000	-4.492033000	5.495929000
H	3.041344000	-3.106236000	5.984049000
S	3.460217000	-5.118496000	7.274825000
O	4.258392000	-4.153598000	8.437244000
H	4.743162000	-4.536503000	7.109203000

Sec-TS$_{iso}$

-2.87701024 Nimag=1 v=-1304.3

C	1.226348000	-3.387840000	7.221488000
C	2.327003000	-3.882132000	6.274095000
N	1.694478000	-2.745516000	8.468960000
H	2.633863000	-3.084892000	8.724471000
H	1.722941000	-1.731623000	8.387073000
H	0.612445000	-2.668155000	6.660404000
C	0.244940000	-4.552450000	7.571015000
O	-0.133117000	-5.364216000	6.750127000
O	-0.143331000	-4.541151000	8.860101000
H	0.387945000	-3.784400000	9.256043000
H	1.899229000	-4.397536000	5.409922000
H	2.994669000	-3.075707000	5.966783000
Se	3.482110000	-5.269612000	7.188870000
O	4.231417000	-4.254682000	8.549875000
H	4.810405000	-4.520843000	7.095592000

Cys-P

-2.99229697 Nimag=0

C	1.239662000	-3.420215000	7.238432000
C	2.312815000	-4.046366000	6.331556000
N	1.749190000	-2.629992000	8.383774000
H	2.700240000	-2.908425000	8.629462000
H	1.753454000	-1.632278000	8.182842000
H	0.619032000	-2.761026000	6.614841000
C	0.260302000	-4.521864000	7.748482000
O	-0.187987000	-5.387487000	7.025725000
O	-0.065509000	-4.388218000	9.051583000
H	0.482853000	-3.608852000	9.355798000
H	1.823892000	-4.678440000	5.579968000
H	2.900915000	-3.279083000	5.815904000
S	3.451481000	-5.203798000	7.181931000
O	4.435517000	-4.114347000	8.080475000
H	5.216091000	-3.918530000	7.528307000

Cys^{-}-P

-2.90442894 Nimag=0

C	1.362862000	-3.030209000	7.264590000
C	2.591976000	-3.287746000	6.373895000
N	1.700737000	-2.371775000	8.561651000
H	2.755150000	-2.406341000	8.686094000
H	1.419067000	-1.392679000	8.547133000
H	0.634801000	-2.408006000	6.709719000
C	0.590907000	-4.344417000	7.583216000
O	0.189725000	-5.126837000	6.735264000
O	0.344188000	-4.481726000	8.903357000
H	0.829547000	-3.649139000	9.266085000
H	2.341899000	-4.022145000	5.598499000
H	2.903054000	-2.341834000	5.900133000
S	4.029153000	-3.947689000	7.341439000
O	4.403310000	-2.701953000	8.315100000

Sec-P

-2.96713264 Nimag=0

C	1.217646000	-3.416784000	7.224400000
C	2.260521000	-4.035434000	6.288240000
N	1.760968000	-2.638301000	8.362464000
H	2.719132000	-2.920949000	8.579413000
H	1.762161000	-1.639015000	8.169307000
H	0.582403000	-2.748175000	6.624421000
C	0.245839000	-4.516796000	7.752244000
O	-0.214666000	-5.384404000	7.038835000
O	-0.056491000	-4.380005000	9.059924000
H	0.506140000	-3.605837000	9.353750000
H	1.768721000	-4.646666000	5.524367000
H	2.881249000	-3.276511000	5.804627000
Se	3.483477000	-5.325624000	7.195184000
O	4.504159000	-4.088814000	8.167928000
H	5.270687000	-3.875609000	7.603977000

Sec^{-}-P

-2.87194672 Nimag=0

C	1.352203000	-3.028929000	7.254405000
C	2.547877000	-3.287087000	6.328607000
N	1.713519000	-2.374384000	8.545421000
H	2.777565000	-2.409687000	8.673827000
H	1.429461000	-1.395908000	8.540536000
H	0.611270000	-2.405440000	6.719221000
C	0.579883000	-4.341635000	7.586951000
O	0.167681000	-5.126426000	6.745790000
O	0.351571000	-4.469627000	8.909062000
H	0.849595000	-3.633136000	9.257763000
H	2.278973000	-3.999315000	5.541673000
H	2.895951000	-2.345167000	5.886605000
Se	4.120141000	-4.044400000	7.357023000
O	4.420687000	-2.651356000	8.439291000

CH_3CdCys-P

-3.55882437 Nimag=0

C	-0.692951000	0.744643000	-0.208686000
C	0.700939000	0.430682000	-0.765448000
N	-0.721803000	1.576453000	1.016272000
H	0.066369000	1.327177000	1.620319000
H	-0.646890000	2.568059000	0.794756000
H	-1.269093000	1.277934000	-0.979244000
C	-1.514765000	-0.544416000	0.067153000
O	-1.410951000	-1.569015000	-0.592891000
O	-2.383442000	-0.423327000	1.083601000
H	-2.172114000	0.475489000	1.473685000
H	0.650326000	-0.005051000	-1.765443000
H	1.313373000	1.337079000	-0.802892000
S	1.651460000	-0.681897000	0.449882000
Cd	0.950074000	-2.866622000	-0.661025000
C	0.578383000	-4.784263000	-1.587593000
H	0.882783000	-4.736299000	-2.636565000
H	1.147940000	-5.558711000	-1.068073000
H	-0.493749000	-4.984167000	-1.514609000
O	3.144784000	-0.408882000	0.238214000

CH_3CdSec-P

-3.51938278 Nimag=0

C	-0.717613000	0.743591000	-0.202799000
C	0.682190000	0.455513000	-0.743056000
N	-0.780784000	1.614573000	0.995694000
H	0.025488000	1.439458000	1.601102000
H	-0.767193000	2.600438000	0.739461000
H	-1.307071000	1.237182000	-0.990943000
C	-1.508855000	-0.558973000	0.104064000
O	-1.386409000	-1.593055000	-0.536324000
O	-2.367150000	-0.439755000	1.130127000
H	-2.178785000	0.475141000	1.494646000
H	0.655552000	-0.044752000	-1.711176000
H	1.280639000	1.366813000	-0.817130000
Se	1.788159000	-0.698553000	0.580682000
Cd	0.971738000	-2.940123000	-0.603643000
C	0.553598000	-4.806142000	-1.619343000
H	0.874329000	-4.720046000	-2.660913000
H	1.094018000	-5.617879000	-1.126423000
H	-0.524761000	-4.975889000	-1.567429000
O	3.393578000	-0.424609000	0.094820000

Cys-RC-SAPE

-5.07247770 Nimag=0

C	0.084386000	0.724532000	-0.292727000
C	0.836460000	0.780167000	-1.633110000
N	0.845906000	1.194324000	0.885331000
H	1.829588000	0.929773000	0.771371000
H	0.798356000	2.207342000	0.982472000
H	-0.817062000	1.349612000	-0.379288000
C	-0.442406000	-0.706223000	0.001770000
O	-0.836716000	-1.470862000	-0.862445000
O	-0.439613000	-1.022257000	1.310442000
H	0.003409000	-0.236605000	1.745651000
H	0.185895000	0.427711000	-2.435600000
H	1.124485000	1.814554000	-1.844232000
S	2.399840000	-0.223178000	-1.592818000
Cd	1.541449000	-2.492778000	-2.095719000
C	0.648796000	-4.290085000	-2.876858000
H	1.295898000	-4.733816000	-3.638715000
H	0.521491000	-4.990677000	-2.046714000
H	-0.326322000	-4.050891000	-3.307153000
O	3.679523000	0.261858000	-4.465914000
O	4.704513000	-0.821577000	-4.423830000
H	4.268900000	-1.500486000	-4.976599000
H	3.241774000	0.134451000	-3.569133000
O	5.501522000	-1.456087000	-1.769423000
H	5.269428000	-1.213767000	-2.697967000
H	5.216156000	-0.685950000	-1.245105000
O	3.709226000	-3.385787000	-1.040348000
H	4.442867000	-2.753937000	-1.283292000
H	4.017287000	-4.274243000	-1.285544000

Sec-RC-SAPE

-5.04642312 Nimag=0

C	-0.028089000	0.788176000	-0.247689000
C	0.871806000	0.973667000	-1.475033000
N	0.536878000	1.246169000	1.041202000
H	1.543948000	1.053369000	1.051877000
H	0.403484000	2.247441000	1.173779000
H	-0.958919000	1.351680000	-0.417803000
C	-0.482902000	-0.687841000	-0.085824000
O	-0.712812000	-1.431096000	-1.025591000
O	-0.622490000	-1.069880000	1.196728000
H	-0.295857000	-0.279396000	1.718627000
H	0.373286000	0.608838000	-2.372980000
H	1.120064000	2.030075000	-1.602585000
Se	2.630121000	0.005139000	-1.268225000
Cd	1.788550000	-2.328143000	-2.004578000
C	1.032785000	-4.046510000	-3.075466000
H	1.840577000	-4.536652000	-3.626896000
H	0.616586000	-4.740860000	-2.339965000
H	0.244709000	-3.733977000	-3.764936000
O	3.598943000	0.111864000	-4.434981000
O	4.149026000	-1.271570000	-4.489872000
H	3.381084000	-1.767267000	-4.839594000
H	3.305704000	0.155855000	-3.473530000
O	5.627211000	-1.900860000	-2.156293000
H	5.151678000	-1.650837000	-2.983696000
H	5.895515000	-1.058073000	-1.752037000
O	3.799571000	-3.362692000	-0.727633000
H	4.548299000	-2.884082000	-1.176660000
H	3.946275000	-4.307417000	-0.905847000

CH_3CdCys-RC-SAPE

-5.07247770 Nimag=0

C	0.084386000	0.724532000	-0.292727000
C	0.836460000	0.780167000	-1.633110000
N	0.845906000	1.194324000	0.885331000
H	1.829588000	0.929773000	0.771371000
H	0.798356000	2.207342000	0.982472000
H	-0.817062000	1.349612000	-0.379288000
C	-0.442406000	-0.706223000	0.001770000
O	-0.836716000	-1.470862000	-0.862445000
O	-0.439613000	-1.022257000	1.310442000
H	0.003409000	-0.236605000	1.745651000
H	0.185895000	0.427711000	-2.435600000
H	1.124485000	1.814554000	-1.844232000
S	2.399840000	-0.223178000	-1.592818000
Cd	1.541449000	-2.492778000	-2.095719000
C	0.648796000	-4.290085000	-2.876858000
H	1.295898000	-4.733816000	-3.638715000
H	0.521491000	-4.990677000	-2.046714000
H	-0.326322000	-4.050891000	-3.307153000
O	3.679523000	0.261858000	-4.465914000
O	4.704513000	-0.821577000	-4.423830000
H	4.268900000	-1.500486000	-4.976599000
H	3.241774000	0.134451000	-3.569133000
O	5.501522000	-1.456087000	-1.769423000
H	5.269428000	-1.213767000	-2.697967000
H	5.216156000	-0.685950000	-1.245105000
O	3.709226000	-3.385787000	-1.040348000
H	4.442867000	-2.753937000	-1.283292000
H	4.017287000	-4.274243000	-1.285544000

CH_3CdSec-RC-SAPE

-5.04642312 Nimag=0

C	-0.028089000	0.788176000	-0.247689000
C	0.871806000	0.973667000	-1.475033000
N	0.536878000	1.246169000	1.041202000
H	1.543948000	1.053369000	1.051877000
H	0.403484000	2.247441000	1.173779000
H	-0.958919000	1.351680000	-0.417803000
C	-0.482902000	-0.687841000	-0.085824000
O	-0.712812000	-1.431096000	-1.025591000
O	-0.622490000	-1.069880000	1.196728000
H	-0.295857000	-0.279396000	1.718627000
H	0.373286000	0.608838000	-2.372980000
H	1.120064000	2.030075000	-1.602585000
Se	2.630121000	0.005139000	-1.268225000
Cd	1.788550000	-2.328143000	-2.004578000

C	1.032785000	-4.046510000	-3.075466000
H	1.840577000	-4.536652000	-3.626896000
H	0.616586000	-4.740860000	-2.339965000
H	0.244709000	-3.733977000	-3.764936000
O	3.598943000	0.111864000	-4.434981000
O	4.149026000	-1.271570000	-4.489872000
H	3.381084000	-1.767267000	-4.839594000
H	3.305704000	0.155855000	-3.473530000
O	5.627211000	-1.900860000	-2.156293000
H	5.151678000	-1.650837000	-2.983696000
H	5.895515000	-1.058073000	-1.752037000
O	3.799571000	-3.362692000	-0.727633000
H	4.548299000	-2.884082000	-1.176660000
H	3.946275000	-4.307417000	-0.905847000

Cys-TS-SAPE

-4.45331012 Nimag=1 v=-189.5

C	1.197483000	-3.246000000	7.434856000
C	2.477150000	-3.275857000	6.582250000
N	1.362408000	-2.807871000	8.837720000
H	2.242679000	-3.155684000	9.224154000
H	1.361299000	-1.792522000	8.919976000
H	0.503385000	-2.542754000	6.952603000
C	0.460151000	-4.618288000	7.398697000
O	0.411916000	-5.318613000	6.411530000
O	-0.155318000	-4.930141000	8.564489000
H	0.090595000	-4.190476000	9.184291000
H	2.258112000	-3.622842000	5.570909000
H	2.933833000	-2.283600000	6.540705000
S	3.751938000	-4.410537000	7.285915000
H	3.583156000	-5.448343000	6.284930000
O	6.751526000	-3.566408000	4.683277000
O	5.305676000	-3.851065000	5.951097000
H	5.978629000	-3.593157000	6.611338000
H	6.255297000	-2.887808000	4.188612000
O	5.973904000	-5.980948000	3.968324000
H	6.303676000	-5.036428000	4.192868000
H	6.086884000	-6.085289000	3.009817000
O	3.561347000	-6.224843000	4.973421000
H	4.497284000	-6.177284000	4.562997000
H	3.335727000	-7.162168000	5.097322000

Sec-TS-SAPE

-4.42402827 Nimag=1 v=-93.3

C	1.103494000	-3.161343000	7.429600000
C	2.443190000	-2.937917000	6.715939000
N	1.092936000	-2.882061000	8.883613000
H	1.987786000	-3.151664000	9.300437000
H	0.937162000	-1.894302000	9.077783000
H	0.365123000	-2.491326000	6.963921000
C	0.546254000	-4.594963000	7.174969000
O	0.627819000	-5.161729000	6.106534000
O	-0.077750000	-5.128260000	8.251834000
H	0.037243000	-4.443591000	8.968068000
H	2.380687000	-3.213644000	5.662962000
H	2.768218000	-1.900117000	6.816589000
Se	3.909701000	-4.071863000	7.484824000
H	3.664574000	-5.156841000	6.408680000
O	6.762083000	-3.624583000	4.408555000
O	5.572534000	-3.656905000	5.714927000
H	6.187962000	-3.230945000	6.344983000
H	6.218042000	-3.058479000	3.827837000
O	6.000158000	-6.241724000	4.122080000
H	6.331998000	-5.297514000	4.180283000
H	6.195002000	-6.532208000	3.216126000

O	3.460839000	-5.857447000	4.954481000
H	4.363102000	-6.135878000	4.618087000
H	2.882854000	-6.637240000	5.002797000

CH_3CdCys-TS-SAPE

-5.06074554 Nimag=1 v=-143.4

C	-0.235266000	0.838617000	-0.863246000
C	1.232427000	0.615765000	-1.247785000
N	-0.472972000	1.557181000	0.409928000
H	0.245391000	1.296193000	1.090960000
H	-0.426837000	2.566701000	0.280762000
H	-0.701141000	1.429079000	-1.666627000
C	-1.040768000	-0.485971000	-0.832557000
O	-0.814157000	-1.435967000	-1.567973000
O	-2.036021000	-0.489884000	0.069170000
H	-1.912341000	0.373651000	0.563896000
H	1.305182000	0.160795000	-2.236743000
H	1.764450000	1.570090000	-1.274713000
S	2.147494000	-0.430929000	-0.023209000
Cd	1.449989000	-2.686445000	-0.884634000
C	0.974601000	-4.779346000	-1.085787000
H	0.370143000	-4.894256000	-1.989596000
H	1.894297000	-5.364370000	-1.172332000
H	0.404510000	-5.115821000	-0.216686000
O	3.982450000	0.012255000	-1.218435000
O	5.249687000	0.565011000	-2.485713000
H	5.975437000	-0.015675000	-2.189119000
H	3.889345000	-0.851167000	-1.675298000
O	3.542006000	-0.140451000	-4.319277000
H	4.293419000	0.161759000	-3.688016000
H	3.922192000	-0.167517000	-5.212009000
O	2.876383000	-2.281026000	-2.952995000
H	3.078053000	-1.517791000	-3.592828000
H	3.136146000	-3.100479000	-3.404341000

CH_3CdSec-TS-SAPE

-5.03965155 Nimag=1 v=-82.1

C	-0.207797000	1.077700000	-0.962514000
C	1.293815000	0.889183000	-1.178157000
N	-0.606450000	1.755557000	0.293564000
H	0.032721000	1.474681000	1.043889000
H	-0.554023000	2.768877000	0.201008000
H	-0.593329000	1.684239000	-1.797506000
C	-0.988541000	-0.258477000	-1.060699000
O	-0.669811000	-1.180346000	-1.797172000
O	-2.075410000	-0.304802000	-0.271310000
H	-2.019755000	0.546260000	0.257118000
H	1.496000000	0.417523000	-2.138891000
H	1.813712000	1.848514000	-1.144640000
Se	2.164655000	-0.219903000	0.259984000
Cd	1.442098000	-2.499193000	-0.752232000
C	0.956835000	-4.565196000	-1.150976000
H	0.447881000	-4.602646000	-2.118171000
H	1.869970000	-5.166550000	-1.184383000
H	0.293715000	-4.950079000	-0.372224000
O	4.209745000	0.266761000	-1.225974000
O	5.282616000	0.736254000	-2.477800000
H	6.066578000	0.240664000	-2.173323000
H	3.999528000	-0.624812000	-1.588431000
O	3.473128000	0.031512000	-4.328434000
H	4.238291000	0.345559000	-3.759238000
H	3.794209000	0.027554000	-5.244660000
O	3.094982000	-2.101366000	-2.762735000
H	3.150371000	-1.405515000	-3.483630000
H	3.369060000	-2.941129000	-3.166574000

Cys-PC-SAPE

-4.56192625 Nimag=0

C	1.364503000	-3.200530000	7.503181000
C	2.030475000	-3.945983000	6.335521000
N	2.288185000	-2.689138000	8.543698000
H	3.168980000	-3.208585000	8.535151000
H	2.501039000	-1.703372000	8.406329000
H	0.810725000	-2.349124000	7.082544000
C	0.272958000	-4.098726000	8.162316000
O	-0.522331000	-4.749482000	7.515994000
O	0.280402000	-4.044610000	9.510718000
H	1.060985000	-3.451802000	9.718135000
H	1.256858000	-4.361751000	5.679357000
H	2.676677000	-3.281844000	5.752253000
S	3.011366000	-5.412763000	6.852219000
H	3.503224000	-6.139520000	4.786863000
O	6.077608000	-3.778228000	5.751278000
O	4.359526000	-4.712380000	7.581453000
H	5.001724000	-4.417785000	6.862166000
H	6.993444000	-3.839574000	6.070910000
O	5.965086000	-4.979817000	3.332370000
H	6.080762000	-4.196036000	4.840769000
H	5.828796000	-4.403809000	2.561610000
O	3.683201000	-6.369445000	3.835661000
H	5.139123000	-5.533154000	3.410410000
H	3.695423000	-7.341463000	3.801187000

Sec-PC-SAPE

-4.53432981 Nimag=0

C	1.348229000	-3.185885000	7.498237000
C	1.941264000	-3.976449000	6.328342000
N	2.326972000	-2.688398000	8.494027000
H	3.186746000	-3.244834000	8.470875000
H	2.575234000	-1.716266000	8.322915000
H	0.811534000	-2.321352000	7.079970000
C	0.247570000	-4.025254000	8.217485000
O	-0.586737000	-4.671298000	7.616480000
O	0.299027000	-3.928034000	9.561798000
H	1.109355000	-3.359989000	9.724340000
H	1.145813000	-4.367975000	5.686825000
H	2.635721000	-3.375253000	5.736577000
Se	2.935591000	-5.610116000	6.917367000
H	3.538882000	-6.242595000	4.694912000
O	6.038496000	-3.763203000	5.789959000
O	4.400001000	-4.793666000	7.684735000
H	4.999069000	-4.487164000	6.941049000
H	6.959090000	-3.779907000	6.101054000
O	5.976376000	-4.902663000	3.326014000
H	6.058630000	-4.158155000	4.871731000
H	5.823023000	-4.305055000	2.575163000
O	3.748789000	-6.424011000	3.740883000
H	5.173847000	-5.490511000	3.375204000
H	3.836219000	-7.390891000	3.676156000

CH_3CdCys-PC-SAPE

-5.14375742 Nimag=0

C	0.427459000	0.914647000	-0.861969000
C	1.777329000	0.667813000	-0.177649000
N	-0.653415000	1.408093000	0.030911000
H	-0.439365000	1.228678000	1.012411000
H	-0.799073000	2.409643000	-0.078472000
H	0.628488000	1.657959000	-1.645695000
C	-0.086893000	-0.342721000	-1.611029000
O	0.609363000	-1.034429000	-2.347234000
O	-1.380973000	-0.606619000	-1.400391000
H	-1.659873000	0.116514000	-0.754543000
H	2.522476000	0.403890000	-0.927605000
H	2.102956000	1.573618000	0.341441000
S	1.721569000	-0.727445000	1.036043000
Cd	1.924782000	-3.034586000	-0.929940000
C	0.252295000	-4.365975000	-0.975448000
H	0.394497000	-5.116318000	-1.756400000
H	0.145435000	-4.853342000	-0.003412000
H	-0.627241000	-3.756739000	-1.195577000
O	3.353572000	-0.889735000	1.358377000
O	2.514943000	2.346082000	-2.806000000
H	2.940205000	1.464713000	-2.970848000
H	3.725408000	-1.347844000	0.529040000
O	3.294836000	-0.306585000	-3.152977000
H	2.801123000	2.908257000	-3.544082000
H	2.351999000	-0.573503000	-3.139307000
O	3.800779000	-2.041535000	-0.985924000
H	3.681801000	-0.869911000	-2.435298000
H	4.540039000	-2.663685000	-1.091018000

CH_3CdSec-PC-SAPE

-5.11572090 Nimag=0

C	0.415608000	0.933086000	-0.886579000
C	1.744788000	0.705705000	-0.166473000
N	-0.670468000	1.464324000	-0.019130000
H	-0.403258000	1.456032000	0.965157000
H	-0.911672000	2.421787000	-0.267171000
H	0.635080000	1.640894000	-1.698527000
C	-0.091628000	-0.361686000	-1.574244000
O	0.591074000	-1.046645000	-2.328693000
O	-1.357372000	-0.675209000	-1.275031000
H	-1.632150000	0.064932000	-0.645847000
H	2.508627000	0.389971000	-0.874430000
H	2.071059000	1.617433000	0.337126000
Se	1.691975000	-0.773282000	1.202774000
Cd	1.933629000	-3.073521000	-0.956187000
C	0.273264000	-4.420240000	-0.991047000
H	0.385113000	-5.127752000	-1.815833000
H	0.216442000	-4.958342000	-0.041780000
H	-0.621209000	-3.810696000	-1.138091000
O	3.504381000	-0.928100000	1.393262000
O	2.516833000	2.376187000	-2.790962000
H	2.927497000	1.487065000	-2.955825000
H	3.787156000	-1.370240000	0.529223000
O	3.255819000	-0.276747000	-3.153520000
H	2.823452000	2.936900000	-3.521919000
H	2.313518000	-0.545463000	-3.139289000
O	3.784020000	-2.046897000	-1.039962000
H	3.645390000	-0.853867000	-2.446900000
H	4.527552000	-2.656693000	-1.182695000
